# Anatomical and Molecular Foundations of the Gut–Brain Axis in Alzheimer’s Disease: From Molecular Mechanisms to Therapeutic Translation

**DOI:** 10.3390/nu18213591

**Published:** 2026-10-29

**Authors:** Bhagath Kumar Potu, Abirami Arulkandan, Prarthana Kalerammana Gopalakrishna, Amardev Singh Thanu, Noor Azzizah Omar, Wael Amin Nasr El-Din, Thirupathirao Vishnumukkala

**Affiliations:** 1 Department of Anatomy, College of Medicine and Health Sciences, Arabian Gulf University, Manama P.O. Box 26671, Bahrain; potubk@agu.edu.bh (B.K.P.); waela@agu.edu.bh (W.A.N.E.-D.); 2 School of Medicine, IMU University, 126, Jln Jalil Perkasa 19, Bukit Jalil, Kuala Lumpur 57000, Malaysia; abilotsalurves@gmail.com; 3 Physiology Discipline, Human Biology Department, IMU University, 126, Jln Jalil Perkasa 19, Bukit Jalil, Kuala Lumpur 57000, Malaysia; prarthana@imu.edu.my; 4 Department of Pre-clinical Sciences, M. Kandiah Faculty of Medicine and Health Sciences, University Tunku Abdul Rahman, Kajang 43000, Selangor, Malaysia; amardev@utar.edu.my; 5 Department of Medical Sciences, Faculty of Medicine and Health Sciences, Universiti Sains Islam Malaysia, Bandar Baru Nilai, Nilai 71800, Negeri Sembilan, Malaysia; azzizah.omar@usim.edu.my; 6 Anatomy Discipline, Human Biology Department, IMU University, 126, Jln Jalil Perkasa 19, Bukit Jalil, Kuala Lumpur 57000, Malaysia

**Keywords:** Alzheimer’s disease, microbiota–gut–brain axis, gut dysbiosis, fecal microbiota transplantation, probiotics, prebiotics, diet

## Abstract

Alzheimer’s disease (AD) is a complex disease that involves systemic, vascular, and metabolic components affecting the brain, in addition to amyloid-beta (Aβ) and tau pathology. The microbiota–gut–brain axis, a bidirectional communication network between the gastrointestinal tract and the central nervous system, involving neural, immune, endocrine, and metabolic pathways, has arisen as a mechanistically plausible and clinically relevant contributor to AD pathogenesis among these systemic contributors. This review integrates information from human observational research, preclinical transgenic and germ-free models, and clinical trials to assess how gut dysbiosis, intestinal barrier dysfunction, and microbial metabolites converge on amyloidogenic and tau-associated pathways. In preclinical models, disruption of intestinal barrier integrity is proposed to allow translocation of lipopolysaccharide and bacterial amyloids into the systemic circulation, activating Toll-like receptor and NLRP3 inflammasome signaling that may prime microglia and astrocytes towards a chronic neuroinflammatory state. Further, dysbiosis-mediated changes in short-chain fatty acid, tryptophan–kynurenine, and bile acid metabolism may influence amyloid precursor protein processing, microglial clearance capability, and tau phosphorylation. Preclinical germ-free and fecal-transfer investigations provide fundamental evidence for these pathways in rodent models, while human case–control and Mendelian randomization data indicate converging yet diverse associations that have not yet established underlying directionality in human disease. Therapeutic modulation of the axis has shown promising early phase and observational signals using probiotics, synbiotics, dietary pattern interventions, fecal microbiota transplantation and pharmacological targeting of the GLP-1 pathway; however, the phase 3 EVOKE and EVOKE+ trials of oral semaglutide did not meet their primary cognitive endpoint, and heterogeneity of trials, short follow-up, and inconsistent reporting of biomarkers currently preclude definitive conclusions. We conclude that the microbiota-gut–brain axis is a biologically plausible and therapeutically tractable component of AD pathophysiology, but translation from mechanistic plausibility to clinical utility will depend upon longitudinal, biomarker-anchored and adequately powered interventional trials in genetically and microbiologically diverse populations. Because diet is among the most readily modifiable determinants of gut microbiome composition and function, this review prioritizes dietary patterns, prebiotic fibers, and probiotic or synbiotic supplementation as practical strategies for investigating the microbiota–gut–brain axis in AD. Their disease-modifying efficacy remains unproven; therefore, mechanistic and pharmacological approaches are discussed as complementary avenues for informing future research.

## 1. Introduction

Alzheimer’s disease (AD) is the leading cause of dementia worldwide and a key contributor to impairment in people over 65 years of age. The Global Burden of Disease Study estimates that there were 57 million people with dementia worldwide in 2019, and this number is expected to increase to 153 million by 2050, mainly in low- and middle-income settings, where diagnostic capacity and care services are very limited [1]. Recent report of the Lancet Standing Commission on Dementia Prevention estimated that roughly 45% of dementia cases worldwide could be prevented in theory by modifying 14 risk factors across the lifespan, such as hearing loss, hypertension, diabetes, obesity, depression, smoking, physical inactivity, social isolation, traumatic brain injury, air pollution, untreated vision impairment and high low-density lipoprotein (LDL) cholesterol [2]. This approach is important for the present analysis as it reconceptualizes AD as a complex illness influenced by systemic, vascular and metabolic variables, not simply intrinsic brain pathology. However, the classic neuropathological feature of AD, which is the presence of extracellular amyloid-beta (Aβ) plaques and intracellular hyperphosphorylated tau that give rise to neurofibrillary tangles, has dominated the treatment landscape for almost two decades [3].

The main hypothesis of the pathogenesis of AD, the amyloid cascade hypothesis, proposes that the accumulation of Aβ is the main driver of synapse loss and neurodegeneration and has guided most of the disease-modifying drug development. Recent regulatory endorsements of anti-Aβ monoclonal antibodies provide a partial affirmation of this method. In the CLARITY AD experiment, lecanemab reduced clinical decline on the CDR-SB by around 27% relative to placebo over 18 months in early symptomatic AD [4], while donanemab had a similar effect magnitude in the TRAILBLAZER-ALZ 2 study [5]. The first drug in this class, aducanumab, received accelerated FDA approval in 2021 amid considerable controversy over its evidentiary basis and was withdrawn in 2024 [6]. These therapies have two limitations that provide the basis for this review. The absolute therapeutic benefit is limited and does not arrest disease progression; patients on treatment continue to decline, albeit more slowly. Second, the class has been connected to amyloid-related imaging abnormalities (ARIAs), including vasogenic edema (ARIA-E) and microhemorrhages or superficial siderosis (ARIA-H), at clinically meaningful rates in both trials [4,5]. This limited efficacy and considerable risk has strengthened the belief that AD is not just due to amyloid accumulation.

Tau pathology, neuroinflammation, cerebrovascular dysfunction, mitochondrial dysfunction, immunosenescence, and metabolic dysfunction all contribute, with the relative importance of each varying between patients and stages of the disease. The move towards a multifactorial view of AD has widened the range of possible therapeutic targets to include systemic mechanisms, such as the bidirectional interaction between the gastrointestinal tract and the central nervous system. The microbiota–gut–brain axis is a system of neuronal, immunological, endocrine, and metabolic routes that allow the gut microbiota and host organs to continuously influence and interact with each other [7]. Microbial metabolites such as short-chain fatty acids (SCFAs) influence microglial maturation and blood–brain barrier integrity in animal models [8]. Also, peripheral inflammation from decreased intestinal barrier function has been associated with cerebral amyloidosis in cognitively challenged people [9]. The nature of such interactions, causative, consequential, or bidirectional, in human AD is unclear.

Several recent reviews have described gut microbiota dysbiosis in AD and mild cognitive impairment, and others have highlighted diet-mediated modulation of the gut–brain axis, particularly via the Mediterranean diet. This review builds on but extends that work by (i) integrating anatomical, molecular, and clinical-trial evidence into a single evidence-weighted narrative framework rather than focusing primarily on microbiome composition or dietary patterns in isolation; and (ii) situating nutrition-based strategies within a broader translational landscape that includes probiotic, synbiotic, fecal microbiota transplantation, and pharmacological interventions targeting the kynurenine and GLP-1 pathways. In doing so, we aim to provide a more mechanistically grounded and clinically actionable synthesis for advancing gut-targeted therapies in AD.

We also discuss the methodological heterogeneity, constraints of cross-sectional design, and the mismatch between mechanistic plausibility in rats and therapeutic applicability in people. The subsequent parts discuss the molecular processes that link gastrointestinal signals to amyloid and tau disease, therapeutic manipulation of the axis, and the present status of clinical trials and conclude with the limitations of the field and a perspective on future approaches.

## 2. Methodology

**Figure 1:**
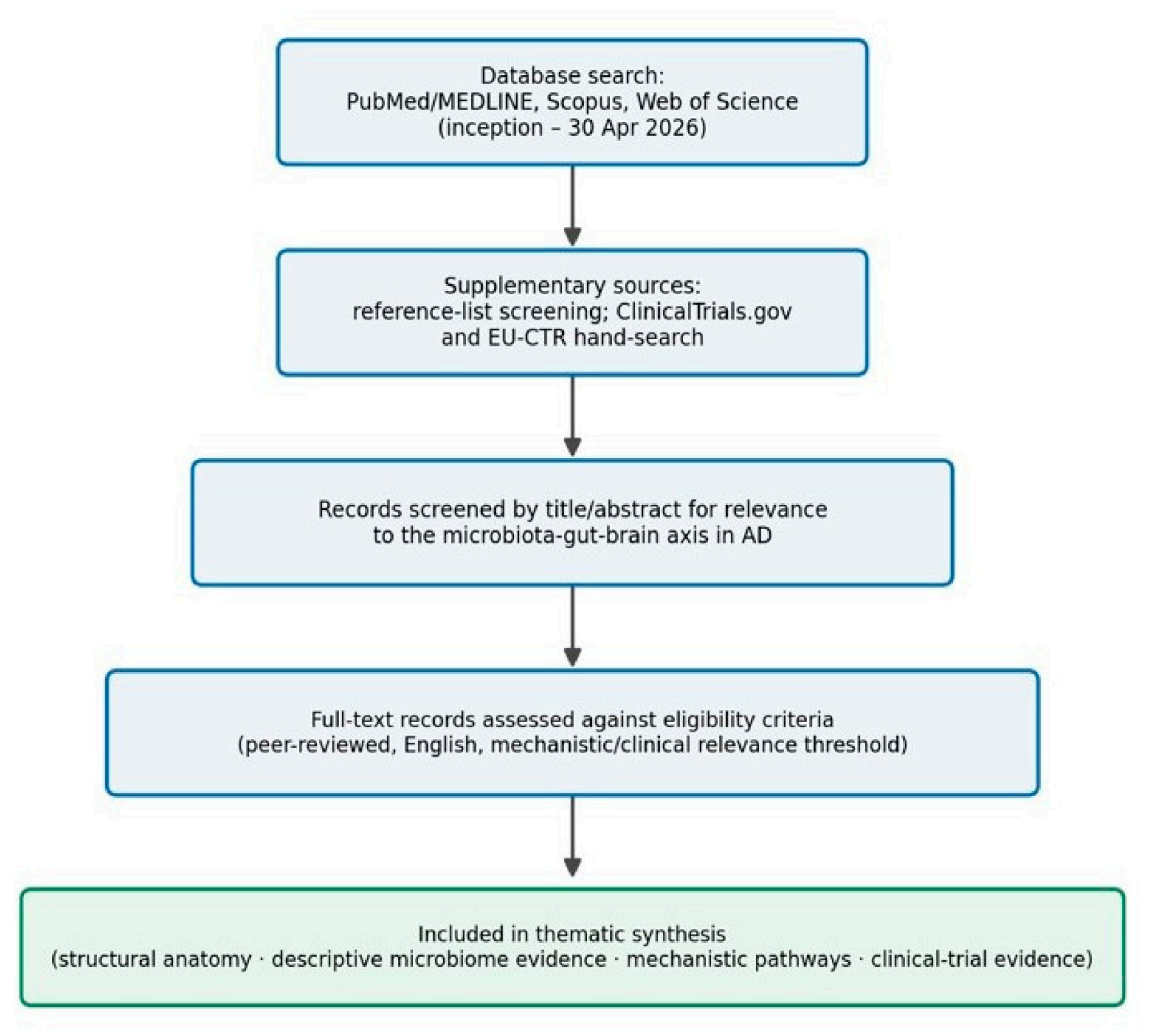
Literature-identification and thematic-synthesis workflow for the structured narrative review. Searches were conducted in PubMed/MEDLINE, Scopus, and Web of Science from database inception to 30 April 2026, supplemented by reference-list screening and searches of ClinicalTrials.gov and the EU Clinical Trials Register. The figure does not represent a PRISMA systematic-review flow diagram, because formal record counting, deduplication, and risk-of-bias assessment were not undertaken.

This is a structured narrative review conducted because the broad, rapidly developing nature of this topic, along with significant variation in study designs, populations, and outcome measures, precludes a traditional systematic review or meta-analysis. To identify the literature as completely as feasible within this narrative framework, we searched PubMed/MEDLINE, Scopus, and Web of Science for records published between inception and 30 April 2026. We identified additional records by screening the reference lists of key reviews and hand-searching ClinicalTrials.gov and the EU Clinical Trials Register for relevant interventional studies. Search terms included controlled vocabulary and free-text keywords such as “AD,” “dementia,” “gut microbiota,” “microbiome,” “gut-brain axis,” “microbiota-gut-brain axis,” “dysbiosis,” “intestinal permeability,” “lipopolysaccharide,” “short-chain fatty acids,” “kynurenine pathway,” “bacterial amyloid,” “faecal microbiota transplantation,” “probiotic,” “prebiotic,” “synbiotic,” and “Mediterranean diet” in combination with “amyloid-beta,” “tau,” “neuroinflammation,” and “microglia.” This is a narrative approach instead of a systematic synthesis; hence, no PRISMA-style record counts, formal deduplication methods, or risk-of-bias/quality assessments were conducted; the literature discovery process is outlined in Figure 1.

Eligible records included peer-reviewed original research articles (human observational and interventional studies, and preclinical animal studies), systematic reviews and meta-analyses, and registered clinical trial records, published in English. Both the mechanistic (cellular and animal) and clinical (human cohort, case–control, and trial) literature were included to allow integration across the translational spectrum, consistent with the narrative-synthesis aims of the review. Conference abstracts without peer-reviewed full text, non-English records, and case reports below a pre-specified threshold of mechanistic or clinical informativeness were excluded unless no higher-quality evidence existed on a given mechanism. Studies were synthesized thematically across four domains: (i) structural and functional anatomy of the microbiota–gut–brain axis; (ii) descriptive evidence for microbiome alteration in AD; (iii) molecular mechanisms linking dysbiosis to amyloid and tau pathology; and (iv) therapeutic and clinical-trial evidence for axis-targeted interventions, in order to map mechanistic plausibility against the current state of clinical proof. Strength of evidence was characterized narratively rather than by a formal grading system (for example, GRADE): findings were described according to study type and consistency, distinguishing preclinical (in vitro and animal) evidence, human observational and Mendelian randomization evidence, and human interventional or registered-trial evidence. This categorization reflects the authors’ judgement and is not a validated scoring method.

## 3. Structural and Functional Organization of the Gut–Brain Axis

The gut–brain axis is the two-way communication system between the gastrointestinal tract and the central nervous system. This encompasses neurological, immunological, endocrine, and metabolic pathways. Increasingly, it is recognized that the resident gut microbiota is an active participant in the gut–brain axis, rather than a passive substrate [7]. Disruption at any node of this network, whether due to dysbiosis, intestinal barrier compromise, chronic systemic inflammation, or vagal dysregulation, has been implicated in the pathophysiology of mood, neurodevelopmental, and neurodegenerative disorders; however, the strength of the evidence available varies considerably across these conditions.

**Figure 2:**
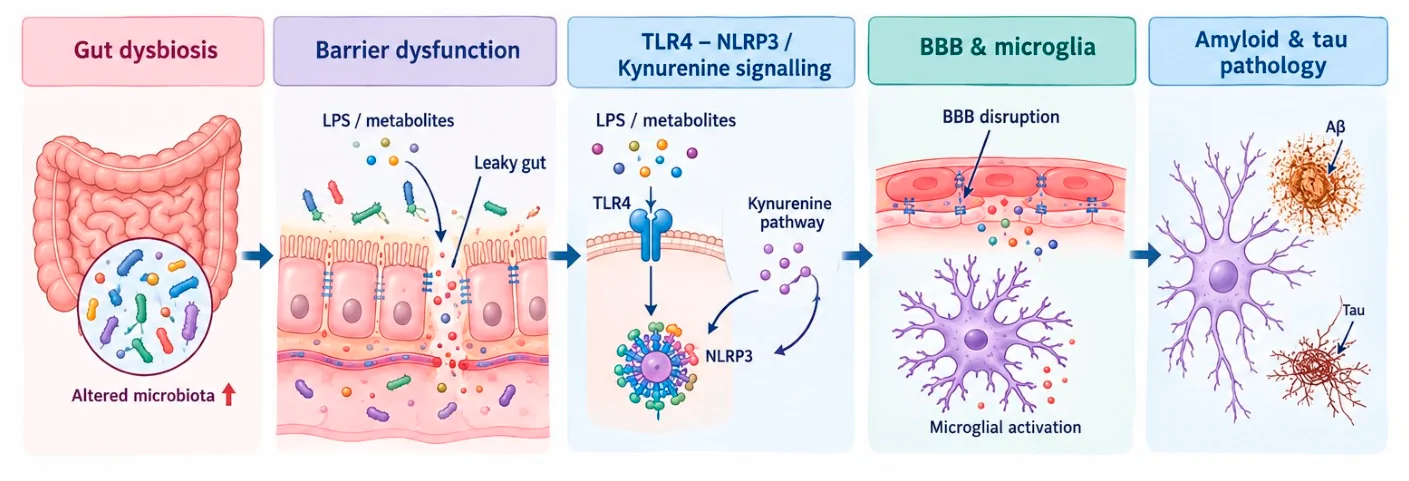
Evidence-informed model of the microbiota–gut–brain axis in AD. Dysbiosis and altered diet may be associated with intestinal-barrier dysfunction and changes in microbial products or metabolites. These signals may influence peripheral immune pathways, blood–brain barrier integrity, microglial responses, and amyloid/tau-related processes. The figure does not imply a proven linear or causal pathway in human AD.

The axis is a complex system and not an isolated anatomical structure. The main components are structured in four interconnected layers (Figure 2).

*Gut microbiota*: Recent estimates suggest that the number of bacterial cells in an adult human is approximately 3.8 × 10^13^, which is of a similar order of magnitude to the number of host cells, rather than the often-quoted tenfold excess [10]. The gut microbiota of a healthy adult consists mainly of Firmicutes and Bacteroidetes, with low representation of Actinobacteria, Proteobacteria, and Verrucomicrobia. Diet, age, geography, exposure to drugs (particularly antibiotics and proton pump inhibitors), comorbidities, and host genetics shape composition. Microbiota manufactures metabolic byproducts, including SCFAs, tryptophan derivatives, secondary bile acids, neurotransmitter precursors, and bacterial cell-wall constituents, which interact with host tissues both locally and systemically [7].

*Intestinal barrier*: Tight junction proteins (claudins, occludin, and zonula occludens-1) anchor a monolayer of intestinal epithelium separating luminal contents from the subjacent lamina propria and coated by inner and outer layers of mucus. Specialized epithelial cells (enterocytes, goblet cells, Paneth cells, and enteroendocrine cells) provide absorptive, secretory, antibacterial, and hormonal functions. The breakdown of barrier integrity, an event also called increased intestinal permeability, causes microbial products and antigens to escape into the systemic circulation and interact with sensors of innate immunity [11].

*Nervous system*: The enteric nervous system (ENS) is made up of about 200–600 million neurons in the gut wall in the myenteric and submucosal plexus. The ENS autonomously controls motility, secretion, and local blood flow, and is continuously monitored by vagal afferents projecting to the nucleus tractus solitarius and then to higher cortical and limbic areas. The vagus nerve is the dominant direct neural connection between the gut and the brain, with afferent fibers around four times more numerous than efferent fibers [7].

*Immune and endocrine systems*: The gut-associated lymphoid tissue (GALT) is the largest immune compartment in the body, consisting of dendritic cells, macrophages, intraepithelial lymphocytes, and Peyer’s patches that interact directly with luminal microorganisms. Enteroendocrine cells release roughly twenty hormones, including cholecystokinin, glucagon-like peptide-1, peptide YY, and serotonin, which have local and systemic effects and integrate into the hypothalamic–pituitary–adrenal (HPA) axis. Microbial metabolites influence the integrity of the blood–brain barrier (BBB), which is anatomically separate from the gut but is the last barrier for gut-derived signals before entering the central nervous system (CNS) [8].

### Communication Pathways

**Figure 3:**
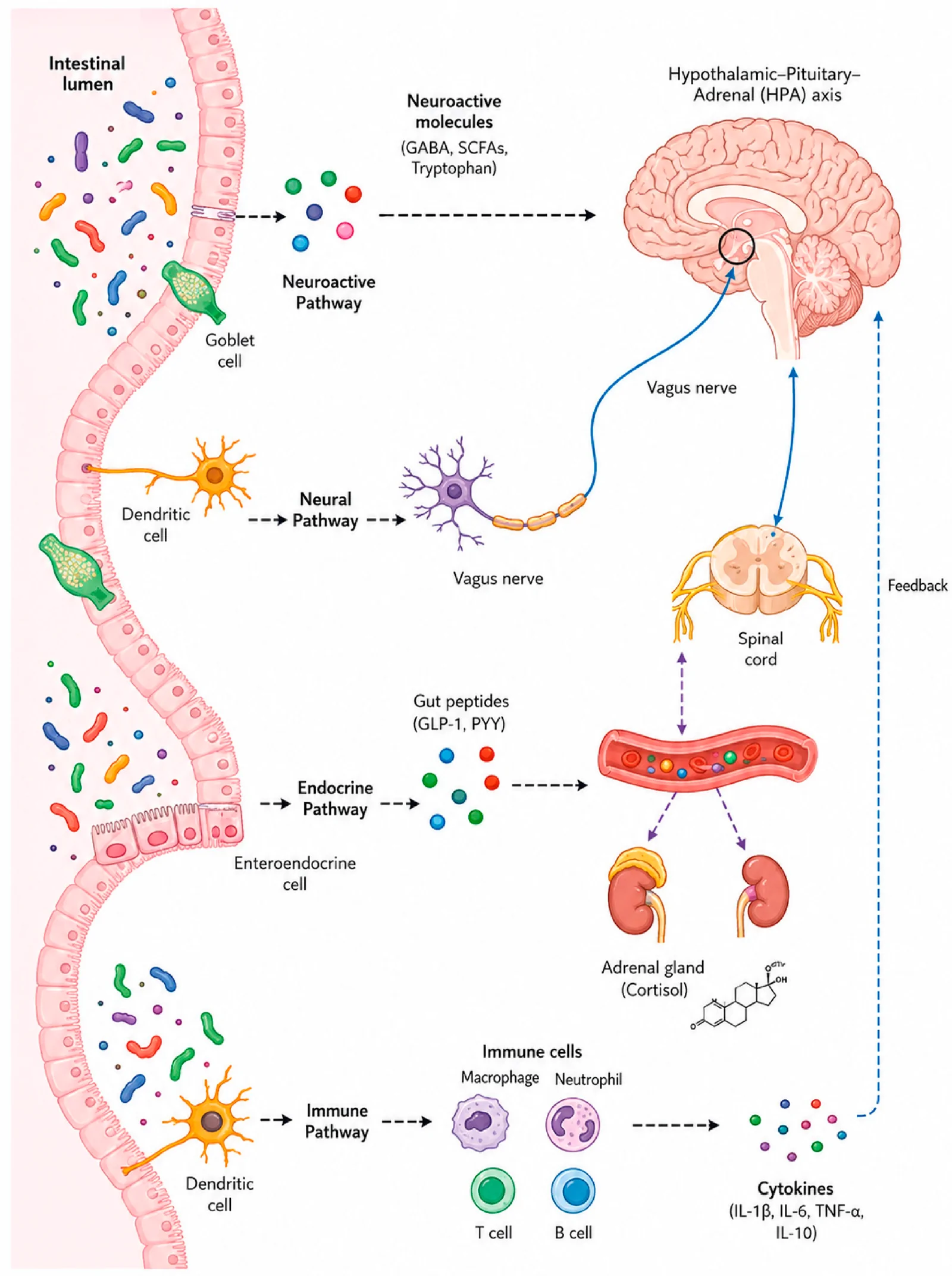
The four principal communication pathways linking the gut microbiota to the brain: The neuroactive pathway (GABA, SCFAs, and tryptophan signaling to the hypothalamic–pituitary–adrenal axis), the neural pathway (vagus nerve afferents from enteric neurons), the endocrine pathway (gut peptides GLP-1 and PYY from enteroendocrine cells), and the immune pathway (macrophages, neutrophils, and T and B cells responding to the intestinal epithelium).

Several partially overlapping mechanisms mediate communication along the microbiota–gut–brain axis. The pathways are not mutually exclusive; a single insult, such as dysbiosis, often triggers all of them together, but for analytical accuracy, it is preferable to analyze them separately. Figure 3 maps each of the four principal communication routes, neuroactive, neural, endocrine, and immune, from the gut microbiota and intestinal epithelium to their respective central and peripheral targets.

*Neural signaling (vagal and enteric pathways)*: Vagal afferents express receptors for microbial metabolites, gut hormones, and inflammatory mediators, allowing the vagus nerve to rapidly signal to nuclei in the brainstem. Electrophysiological and chemogenetic studies in mice have shown that vagotomy can abolish the behavioral and neurochemical effects of specific bacterial strains, demonstrating that the vagal pathway is functionally relevant and is not only anatomical [7]. The ENS is largely a local regulator, but it also transmits signals to the CNS via spinal afferents and is regarded as one of the first sites to be affected in many neurodegenerative illnesses, particularly Parkinson’s disease (PD).

*Immune and inflammatory signaling*: Bacterial lipopolysaccharide (LPS) and peptidoglycan translocation across a compromised intestinal barrier activates Toll-like receptor 4 (TLR4) and TLR2 on peripheral innate immune cells, resulting in higher circulating levels of IL-1β, IL-6, TNF-α, and CXCL2. Chronic low-grade systemic inflammation is associated with peripheral monocyte priming and altered microglial phenotypes in CNS, a state sometimes referred to as microglial priming. In cognitively impaired individuals with brain amyloidosis, increased plasma levels of IL-1β, NLRP3, and CXCL2 have been correlated with a higher prevalence of pro-inflammatory Escherichia/Shigella and a decreased prevalence of anti-inflammatory Eubacterium rectale [9]. In humans, it is not yet known whether peripheral inflammation promotes central neuroinflammation, reflects central neuroinflammation, or both.

*Microbial metabolites and endocrine mediators*: The most researched microbiota-derived signaling molecules are short-chain fatty acids, predominantly acetate, propionate, and butyrate, which are produced by bacterial fermentation of dietary fiber. They improve the integrity of tight junctions; act as histone deacetylase inhibitors; and signal through G-protein-coupled receptors GPR41, GPR43, and GPR109A. Microglia in germ-free mice show immature morphology, reduced responses to immunological activation, and impaired host defense; SCFA supplementation partially restores mature microglial features. Other metabolic pathways include tryptophan-derived metabolites (kynurenine and indoles), secondary bile acids, and trimethylamine-N-oxide (TMAO) [7]. All are tentatively associated with neurodegeneration, but caution is needed, as much of the evidence is preclinical.

The HPA axis integrates information from microbiota with central stress responses. Germ-free mice have an exaggerated corticosterone response to stress that is partially adjusted following colonization early in life, indicating a developmental window during which the microbiota contributes to calibrating stress physiology [7]. Enteroendocrine signaling offers other avenues for microbial composition to affect appetite, mood, and inflammation: about 90% of the body’s peripheral serotonin is made in the enterochromaffin cells of the gut, and gut-derived GLP-1 and peptide YY also interact with both the central and immune systems.

*Amyloids of bacteria*: One conceivable avenue would be microbial production of amyloidogenic proteins. Bacterial curli fibrils secreted by E. coli are structurally similar to human amyloids and can promote cross-seeding of α-synuclein aggregation in rats and C. elegans models and primary TLR2-mediated neuroinflammation [12]. The clinical relevance of bacterial amyloids in Aβ aggregation in human AD is not yet clear and is supported by molecular plausibility rather than direct clinical proof.

Having established the structural and signaling architecture of the axis, the next section turns to the empirical question that follows directly from it: if these pathways are functionally relevant to AD, does the composition of the gut microbiota differ in patients with the disease?

## 4. Alterations of the Gut Microbiome in Alzheimer’s Disease

If the microbiota–gut–brain axis is functionally relevant to AD, the most parsimonious inference is that the composition or function of the gut microbiota will differ between individuals with AD and cognitively intact controls, and that these differences will correlate with disease severity, biomarker load, or risk factors. There are currently substantial data that support this idea in broad terms; however, there is variability in the fingerprints of individual microbes among studies. Research in animal models provides more molecular insight but cannot be directly applied to the heterogeneous, late-onset, and slowly progressive nature of human sporadic AD.

### 4.1. Evidence from Human Studies

Since 2017, case–control studies have been conducted comparing the gut microbiome of AD patients with cognitively normal controls. Vogt et al. reported reduced microbial richness and a distinct beta-diversity profile in AD, with reduced Firmicutes and Bifidobacterium, and increased Bacteroidetes, in a US dataset of 25 AD sufferers and 25 age- and sex-matched controls [13]. Genera with differential abundance are related to cerebral fluid indicators, including the Aβ42/Aβ40 ratio and phosphorylated tau, suggesting a link between microbial composition and core disease pathology, even in a small cohort. Liu et al. expanded on this investigation by examining a Chinese population of 33 patients with AD, 32 individuals with amnestic mild cognitive impairment, and 32 cognitively healthy controls [14]. AD was again linked to a lower abundance of Firmicutes and a higher abundance of Proteobacteria, with a progressive enrichment of Enterobacteriaceae from controls to aMCI to AD. Altered taxonomic proportion was correlated with clinical severity, and a microbiome feature-based discriminant model could distinguish AD from controls with AUC of 0.94. These data support the use of microbial profiles as potential biomarkers, but they do not address the temporal directionality of the association.

Ferreiro et al. evaluated cognitively healthy older adults stratified by amyloid PET status and found compositional differences in the microbiota between Aβ-positive and Aβ-negative subjects [15]. The researchers observed alterations in microbial composition during the preclinical stage of AD; however, the cross-sectional methodology and limited sample size exclude any conclusions on timing or causative relationships. The findings primarily serve to generate hypotheses: they suggest that alterations in composition might transpire before cognitive decline; however, longitudinal studies examining microbiota and biomarkers in the same individual over time are necessary to determine if these changes genuinely precede the onset of disease. The broader literature is, hence, quite diverse.

In a systematic review and meta-analysis of 17 studies, Jemimah et al. reported consistent reductions in alpha diversity in AD relative to controls, but they found limited agreement with respect to specific taxa, with Bacteroidetes reported as both elevated and diminished in AD relative to controls depending on the cohort [16]. A considerable part of this inconsistency is presumably due to methodological issues. The sequencing techniques (16S rRNA versus shotgun metagenomics), targeted variable regions, bioinformatics frameworks, sample sizes (usually 20–100 individuals per group), and rigor of clinical phenotyping (clinical diagnosis versus biomarker-validated AD) vary among the research methodologies.

Confounders intrinsic to dementia research are also poorly controlled across studies. Patients with AD tend to live in institutional settings with communal diets, to be subject to polypharmacy including drugs known to affect the microbiome (e.g., proton pump inhibitors, antibiotics, and antipsychotics), to have constipation and abnormal gastrointestinal transit, and to have differences in physical activity in comparison to cognitively normal individuals. Each of these factors may cause microbiome differences that are not specific to the development of AD. Variations in geography and diet among cohorts further complicated cross-study comparisons. An accurate reading of the human data points to changes in the gut microbiome in AD in terms of diversity and overall compositional structure, that some compositional differences may be detectable prior to clinical cognitive decline, and that specific taxa are associated with biomarkers of pathology. What the human data cannot yet establish is whether these alterations are causally linked to neurodegeneration, whether they are downstream effects of lifestyle and physiological changes associated with AD, or whether they represent a bidirectional process over time. The preclinical literature reviewed next was designed specifically to address this question of causality.

### 4.2. Evidence from Preclinical Models

Preclinical studies have generated compelling evidence that gut microbial composition may causally influence AD-related pathology, whilst also underlining the limits of extrapolating findings from rodent models to human disease. Harach et al. conducted a landmark germ-free study in which they generated APP_SWE_/PS1 (APPPS1) transgenic mice in a germ-free environment and examined cerebral Aβ pathology relative to conventionally raised littermates [17]. The germ-free APPPS1 mice had a marked reduction in cerebral Aβ plaque deposition. Colonization of germ-free APPPS1 mice with microbiota from conventionally raised APPPS1 donors restored amyloid pathology, whereas colonization with microbiota from wild-type donors resulted in only a partial augmentation. This work clearly shows that the presence and composition of the microbiota affect amyloidogenesis in a transgenic model but does not identify the microbial determinants, and is applicable to a familial rather than sporadic form of AD.

Antibiotic alteration of microbiota has yielded broadly consistent consequences. In APPswe/PS1dE9 mice, long-term treatment with broad-spectrum antibiotics led to reduced Aβ plaque deposition, altered microglial morphology, and changes in serum levels of cytokines and chemokines [18]. The same research group further investigated the phenomenon and showed that antibiotic treatment restricted to the early postnatal period led to long-lasting reductions in Aβ pathology in aged mice, suggesting a developmental time window during which microbiota–host interactions can influence subsequent neuroinflammatory responses. Sex-specific effects of various antibiotic regimens have since been reported, complicating any simple narrative.

Further evidence for a causal contribution comes from microbiota-transfer research. Sequential fecal microbiota transfers from wild-type donors to ADLPAPT mice, a triple transgenic model with mutations in APP, PS1, and tau, reduced Aβ plaques and tau tangles, alleviated glial activation, and improved cognitive function [19]. This is one of the first demonstrations that microbiome modification can affect both major proteinopathies of AD in the same animal model. Not all preclinical studies converge on a single conclusion, however. Colombo et al. reported that short-chain fatty acids (SCFAs), normally regarded as beneficial bacterial metabolites, surprisingly increased Aβ plaque deposition when administered to germ-free APP/PS1 mice [20]. SCFA supplementation changed microglial transcriptomes, leading to ApoE activation and reduced microglial Aβ phagocytosis, while increasing recruitment of microglia to plaque areas. This finding emphasizes that microbial metabolites cannot be definitively labeled as protective or deleterious, since the same metabolite may provoke different responses depending on host genotype, developmental stage, and concurrent disease.

Research on bacterial amyloids has indicated a further mechanistic possibility: cross-seeding between microbial and host amyloidogenic proteins. Chen et al. demonstrated that exposure of aged rats and C. elegans to curli-generating Escherichia coli increased neuronal α-synuclein accumulation in the gastrointestinal tract and brain, and induced TLR2-, IL-6-, and TNF-mediated neuroinflammation [12]. The best evidence to date concerns α-synuclein rather than Aβ, and its relevance to human AD remains speculative.

Overall, the preclinical data indicate that experimental manipulation of the microbiota can modulate Aβ accumulation, tau-related pathology, microglial phenotypes, and behavior in selected transgenic and antibiotic-treated mouse models. These findings establish causality only within the experimental systems studied and should not be interpreted as proof that microbiota causes sporadic human AD. The convergence of human associative findings with causal effects observed in selected rodent models is hypothesis-generating, but it does not establish that comparable causal relationships occur in sporadic human AD.

## 5. Mechanistic Pathways Linking the Gut–Brain Axis to AD Pathology

The preceding sections indicate that gut microbial composition and function may differ between individuals with AD and controls, while experimental manipulation of the microbiota can influence AD-related phenotypes in selected animal models. We now turn to the specific molecular mechanisms thought to translate dysbiosis and barrier dysfunction into amyloid and tau pathology in the brain.

### 5.1. Intestinal Barrier Dysfunction and Systemic Inflammation

The human gastrointestinal tract is now broadly recognized as a crucial determinant of systemic and neurological well-being, rather than merely a digestion apparatus. The internal surface, composed of a singular layer of tightly connected epithelial cells that spans a considerable area (~30 m^2^), functions as a highly discerning interface. This barrier facilitates efficient nutrition uptake while maintaining immune tolerance to countless commensal microorganisms and restricting the dissemination of harmful pathogens and poisons [11].

Research suggests that, in AD, dysfunction of the intestinal barrier may occur prior to cognitive symptoms, allowing harmful substances to enter the bloodstream and trigger chronic low-grade inflammation, which is thought to play a crucial role in neurodegeneration [21].

At the molecular scale, the impairment of tight junction proteins, alterations in gut microbiota, and immune system activation synergistically enhance systemic inflammatory signals. Cytokines and microbial substances in the circulatory system might modify the blood–brain barrier, possibly leading to neuroinflammation and phenomena such as amyloid-β buildup and tau hyperphosphorylation [22]. This section explores the mechanistic basis of intestinal barrier impairment and its relationship with systemic inflammation. These pathways provide a biologically plausible link between gut dysfunction and AD-related pathology, although their temporal and causal relevance to human AD remains uncertain.

*Elevated intestinal permeability*: The gastrointestinal (GI) system is an intricate network of organs responsible for the digestion and absorption of nutrients. In optimal health, the intestinal barrier functions as a sophisticated multi-tiered defense mechanism that preserves a fragile equilibrium between host immunity and bacteria acceptance. The mucus layer produced by goblet cells constitutes the outermost aspect of the epithelial surface, serving to physically isolate luminal microorganisms from the epithelial layer. Underneath this exists a solitary stratum of intestinal epithelial cells, securely bound by intercellular junctions that govern permeability [23]. Tight junctions (TJs) constitute an essential component of this system. They consist of transmembrane proteins such as claudins, occludin, and junctional adhesion molecules, and they are anchored within the cell by zonula occludens proteins (ZO-1, ZO-2, and ZO-3), which connect the structure to the underlying actin cytoskeleton. These formations establish an active molecular barrier that permits the influx of nutrients while obstructing the ingress of detrimental chemicals [24]. Occludin is not essential for the establishment of tight junction strands; instead, it plays a crucial regulatory role. The phosphorylation state functions as a toggle that dictates the permeability of the barrier. Moreover, the *C*-terminal coiled-coil domain (OCEL) is crucial for the disruption of barriers produced by TNF-α [25].

In transgenic mouse models of AD, the buildup of Aβ has been detected in both the cerebral tissue and the intestinal epithelium, which is associated with impaired intestinal barrier integrity. Thorough analyses of the intestinal mucosa in AD mice reveal both structural and functional changes, including increased intestinal permeability, inflammatory modifications, and reduced levels of critical tight junction proteins such ZO-1, occludin, and claudin-1. Ultrastructural irregularities, such as expanded paracellular spaces and diminished microvilli, additionally indicate barrier failure. Compensatory goblet and Paneth cell responses may occur early in barrier injury, but sustained dysbiosis is associated with thinning and depletion of the mucus layer. Significantly, studies with transgenic animals (such as APP/PS1 and 3×Tg-AD mice) demonstrate that modifications in the intestinal barrier might occur prior to considerable amyloid accumulation in the brain, implying that intestinal barrier dysfunction may not solely be a consequence of AD but could also promote its early development. Gut physiology and microbial function therefore warrant investigation as potential early therapeutic targets; however, this possibility is currently supported mainly by transgenic-mouse evidence and has not been demonstrated in human AD [26].

In human research, individuals with AD and mild cognitive impairment (MCI) demonstrate markedly elevated serum indicators of intestinal permeability, such as diamine oxidase (DAO), D-lactic acid (DA), and endotoxin (ET), in contrast to cognitively intact controls. A cross-sectional investigation comprising 118 subjects (45 diagnosed with AD, 38 with MCI, and 35 controls) demonstrated a notable inverse relationship between the extent of gut barrier impairment, evaluated using these biomarkers, and cognitive functioning, as reflected by MMSE and MoCA assessments. The research also observed a reduced occurrence of barrier-protective bacteria, including Bacteroides and Faecalibacterium, in patients with AD [27].

Gut microbial dysbiosis, characterized by alterations in the composition, diversity, and metabolic function of gut microbiota that promote signals detrimental to barrier integrity over those that enhance it, significantly contributes to intestinal barrier impairment in AD. This disparity initiates a sequence of molecular and cellular processes that gradually undermine epithelial integrity [28]. This procedure is facilitated by various interrelated factors.

*Diminished butyrate synthesis*: Faecalibacterium prausnitzii and Roseburia intestinalis are advantageous bacteria that synthesize the short-chain fatty acid (SCFA) butyrate, whereas Akkermansia muciniphila produces the SCFAs acetate and propionate and supports mucus-layer integrity. Butyrate serves as the primary energy substrate for colonocytes and contributes to the preservation of barrier integrity by elevating the concentrations of tight junction proteins, including claudin-1, occludin, and ZO-1. It additionally activates the G-protein-coupled receptor GPR109A (HCAR2), triggering signaling pathways that promote the stability of epithelial cells. In AD, reduced levels of fecal SCFAs, especially butyrate, have often been reported and are associated with impaired barrier integrity [29].

*Cytokine-induced impairment of tight junctions*: Dysbiosis cultivates a pro-inflammatory milieu characterized by elevated levels of cytokines, including TNF-α and IL-1β. These cytokines undermine the integrity of tight junctions through unique yet complementary ways. TNF-α activates myosin light-chain kinase (MLCK), resulting in cytoskeletal contraction that separates tight junction proteins. IL-1β enhances NF-κB signaling, leading to elevated levels of claudin-2, a protein that forms pores and heightens permeability, concurrently reducing the production of barrier-forming claudins. Collectively, these phenomena establish a detrimental loop wherein inflammation and barrier impairment intensify each other [30,31].

*Mucus-layer depletion and reduced AhR signaling*: Dysbiosis is frequently linked to weakening of the mucus layer, caused by proliferation of mucin-degrading bacteria and altered goblet cell activity, which makes the epithelial surface more vulnerable to microbial antigens. Dysbiosis also impairs microbial conversion of dietary tryptophan into metabolites such as indole-3-acetic acid and indole-3-propionic acid, which bind to the aryl hydrocarbon receptor (AhR). Reduced AhR activation reduces the generation of IL-22, a cytokine required for epithelial repair and mucus synthesis, further weakening barrier integrity [32].

*Outer membrane vesicles as mediators of remote signaling*: Gram-negative bacteria release outer membrane vesicles (OMVs), a novel and underexplored signaling mechanism. These tiny vesicles contain lipopolysaccharide (LPS), proteins, and nucleic acids, and they can cross the mucus layer to interact directly with intestinal epithelial cells. OMVs can disrupt tight junctions and trigger inflammatory signaling without requiring full bacterial translocation [33]. Some OMVs, such as those from Helicobacter pylori, have been proposed to reach the central nervous system, where they may activate glial cells and exacerbate amyloid pathology, which would represent an efficient channel for gut-derived signals; this remains largely experimental [34].

Most reviews treat intestinal permeability as a binary state, the gut either “leaks” or it does not. A more nuanced and clinically meaningful perspective posits that gut permeability exists as a dynamic continuum comprising at least two distinct pathological states: (1) pore-pathway hyperactivation, driven by elevated claudin-2 expression and characterized by small-ion flux without macromolecular translocation; and (2) leak-pathway failure, involving disassembly of barrier-forming claudins and occludin that facilitates translocation of LPS, bacterial amyloids, and metabolic endotoxins into systemic circulation. In AD, both mechanisms presumably operate; however, leak-pathway failure is the more pathologically significant, as it permits translocation of macromolecules large enough to activate systemic and central immune pathways [35].

**Figure 4:**
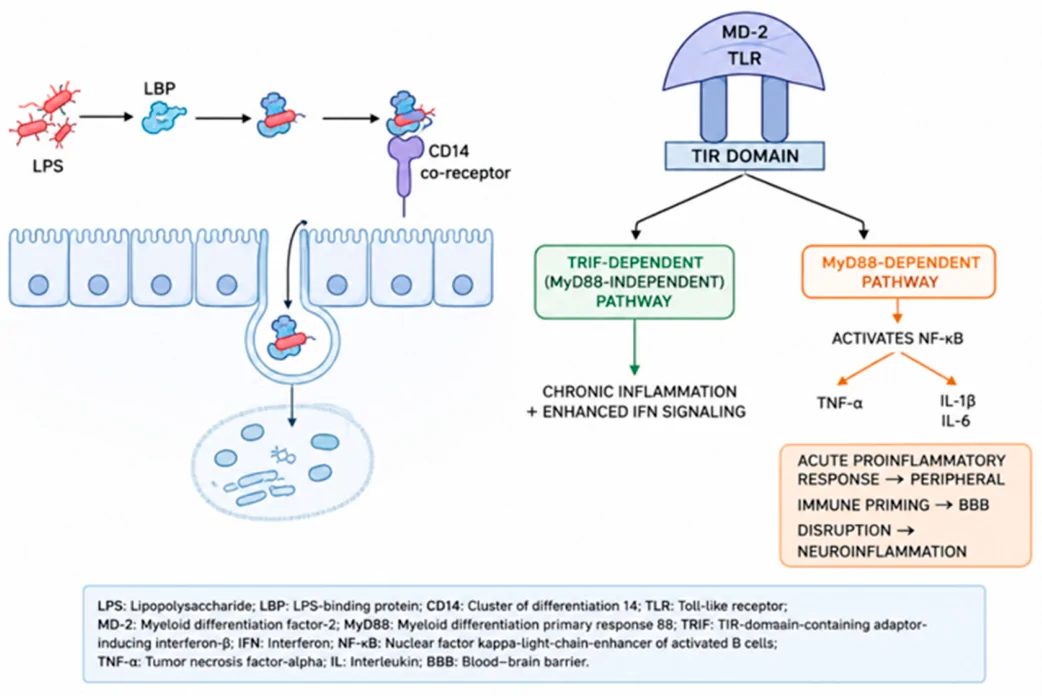
Lipopolysaccharide (LPS) translocation and Toll-like receptor 4 (TLR4) signaling. LPS released from Gram-negative bacteria is bound by LPS-binding protein (LBP) and presented via CD14 to the TLR4–MD-2 receptor complex, activating the TIR domain and bifurcating into the TRIF-dependent (MyD88-independent) pathway, sustaining chronic inflammation and interferon signaling, and the MyD88-dependent pathway, which activates NF-κB and drives TNF-α, IL-1β, and IL-6 production, producing peripheral immune priming, blood–brain barrier disruption, and neuroinflammation.

*Endotoxemia and immune activation*: In AD, breakdown of the intestinal barrier permits lipopolysaccharide (LPS), a key component of Gram-negative bacterial membranes, to enter the bloodstream, a state termed metabolic endotoxemia [36]. LPS is a potent stimulator of innate immunity even at low physiological levels and represents an important link between gut dysfunction and systemic inflammation. Circulating LPS binds LPS-binding protein (LBP) and is presented to CD14, which helps activate the TLR4–MD-2 receptor complex on immune and endothelial cells. This activates two key intracellular pathways: the MyD88-dependent pathway, which activates NF-κB and produces the proinflammatory cytokines TNF-α, IL-1β, and IL-6; and the TRIF-dependent (MyD88-independent) pathway, which sustains chronic inflammation and enhances interferon signaling [37]. Figure 4 illustrates this cascade, from LPS translocation across the epithelium through CD14-mediated TLR4 activation to the divergent MyD88- and TRIF-dependent downstream consequences for neuroinflammation.

Gut dysbiosis disrupts the integrity of the intestinal barrier, resulting in heightened intestinal permeability, commonly known as “leaky gut.” This enables bacterial toxins, including LPS and various microbial-associated molecular patterns (MAMPs), to infiltrate systemic circulation. In experimental epithelial models, LPS can increase the permeability of intestinal tight junctions by activating focal adhesion kinase (FAK) through TLR4, which disrupts the cytoskeletal anchoring of tight junction proteins [37]. This initiates a self-reinforcing positive-feedback loop: circulating LPS further damages the intestinal barrier, increasing gut permeability and allowing more endotoxin into the bloodstream, thereby producing a sustained cycle of endotoxemia that perpetuates systemic inflammation.

### 5.2. Neuroinflammatory Signaling

Peripheral immune priming and BBB compromise: When LPS enters the bloodstream, peripheral immune cells such as monocytes, macrophages, neutrophils, and T-lymphocytes rapidly detect it and mount a strong inflammatory response. LPS primes monocytes and macrophages toward a hyperinflammatory state, making them more readily activated. In AD, this peripheral immune priming is especially important because repeated exposure to Aβ oligomers, which also activate TLR4, produces a stronger inflammatory response than either signal would cause alone.

The proinflammatory cytokines produced during this peripheral response, together with circulating LPS, affect brain endothelial cells, disrupting the blood–brain barrier (BBB) by downregulating the tight junction proteins claudin-5 and occludin. Post-mortem analyses of AD brains demonstrate a specific reduction in cortical claudin-5 and occludin that correlates with Braak neurofibrillary tangle stage and shows an inverse association with synaptic marker abundance, suggesting that loss of BBB tight junctions is a gradual, stage-dependent phenomenon in AD, intimately associated with the severity of the underlying neuropathology [38].

*Neuronal and humoral routes to the brain*: Peripheral immune activation interacts with the central nervous system via several convergent mechanisms that extend beyond direct disruption of the BBB. Circulating pro-inflammatory cytokines can cross the damaged BBB, and activated immune cells such as monocytes and T cells can infiltrate brain tissue directly. In parallel, neural routes, particularly the vagus nerve, convey inflammatory signals from the gut to the brainstem, offering a rapid and direct path for peripheral inflammation to alter CNS function. Circumventricular organs have a barrier that is less robust than the BBB, thus making it easier for peripheral cytokines to reach brain parenchyma without needing to fully breach the BBB. Gut-derived metabolites and neurotransmitter-like molecules affect brain function through both humoral and neuronal routes, reinforcing the bidirectional communication that characterizes the gut–brain axis [39]. Collectively, these pathways provide several possible routes through which peripheral gut-associated signals may influence CNS. The relative contribution of each route, as well as whether these pathways are sufficient to affect AD progression in humans, remains unresolved.

*Cytokines and inflammatory mediators*: Cytokines are small proteins that serve as the body’s inflammatory signaling messengers. Under normal circumstances, they are direct short-term immune activity and then shut off; in AD, they become chronically activated and circulate at elevated levels throughout blood and brain, producing chronic neurotoxicity. In AD, cytokines determine both localized immune activity within the CNS and distal immune signaling originating outside the brain. Pro-inflammatory cytokines such as TNF-α and IL-1β are neurotoxic and drive inflammation by activating microglia and astrocytes, further amplifying cytokine cascades and directly damaging neurons. In AD, microglia and astrocytes produce cytokines like TNF-α, IL-1β, IL-6, IL-2, IL-12, and IFN-γ, which together promote neuroinflammatory cascades [40].

Four pro-inflammatory cytokines are particularly central to this process. Tumor necrosis factor-alpha (TNF-α) functions as a master switch for neuroinflammation: high levels in the AD brain impair neuronal communication by disrupting synapses, increase amyloid production, and initiate downstream inflammatory cascades, while also damaging the blood–brain barrier and allowing further peripheral pro-inflammatory signals to enter the brain [41]. Interleukin-1 beta (IL-1β) is among the first cytokines activated in AD and one of the most potent drivers of inflammation; it stimulates microglia to produce additional inflammatory mediators, contributes to tau hyperphosphorylation, and inhibits the neuronal plasticity mechanisms underlying memory formation [42]. Interleukin-6 (IL-6) acts as a double-edged sword, reducing inflammation and aiding amyloid clearance under some conditions, but promoting chronic neuroinflammation when persistently elevated; in AD brains, IL-6 activates JAK-STAT signaling in neurons and glial cells, feeding neuroinflammation and neuronal dysfunction [43]. Interferon-gamma (IFN-γ) heightens microglial reactivity and enhances antigen-presenting capacity, attracting additional immune cells to the brain and worsening inflammation [44].

This escalating inflammatory burden is itself exacerbated by cytokine-driven amplification of amyloid and tau pathology. When microglia become activated, they release TNF-α, IL-1β, and IL-6 into brain tissue; these cytokines increase amyloid production by upregulating BACE1 and contribute to tau phosphorylation by activating GSK-3β. Neuroinflammation thus sits at the center of AD pathophysiology, intersecting amyloid-beta plaque accumulation, tau tangle formation, and neuronal degeneration [45].

Microglial NLRP3 activation and pyroptosis: When LPS and proinflammatory cytokines breach the BBB, they activate microglia in a two-step process. First, priming: LPS activates microglial TLR4, which activates NF-κB and upregulates transcription of NLRP3 and precursors of pro-inflammatory cytokines [46]. Second, activation: A second signal, such as Aβ oligomers, extracellular ATP from damaged neurons, mitochondrial reactive oxygen species, or potassium efflux, triggers assembly of the NLRP3 inflammasome. NLRP3 oligomerizes, recruits the adaptor protein ASC (apoptosis-associated speck-like protein) and activates caspase-1. Caspase-1 then cleaves pro-IL-1β and pro-IL-18 into their mature, active forms and cleaves gasdermin D (GSDMD), releasing its *N*-terminal fragment, which forms pores in the plasma membrane and causes pyroptosis, an inflammatory form of cell death that releases intracellular contents and amplifies local inflammatory signaling [47].

In experimental AD models, activation of the NLRP3–pyroptosis axis has been associated with worsening amyloid-related pathology, neuroinflammation and cognitive performance. Whether this pathway mediates disease progression in human AD, as well as the extent to which it is driven by gut-derived signals, remains to be established. In APP/PS1 transgenic mice, peripheral LPS exposure enhances NLRP3 activation in the cortex and hippocampus, impairs microglial clearance of Aβ, disturbs glymphatic function, and worsens cognitive performance. Together, these findings are consistent with an underlying relationship between endotoxemia and AD-related brain pathology in this transgenic mouse model [48]; whether the same relationship holds in human AD remains to be established. Pyroptotic microglia release ASC specks, which bind to amyloid-β and act as pro-inflammatory “seeds” that accelerate Aβ aggregation and plaque formation, linking neuroinflammation directly to protein aggregation. NLRP3 activation also worsens tau pathology: activated microglia release IL-1β, which activates GSK-3β, the kinase responsible for tau hyperphosphorylation. These findings provide a model in which gut-associated inflammation could contribute to amyloid and tau-related pathology through amplification of neuroinflammatory signaling. Direct evidence that this sequence drives human AD is currently lacking [49].

Beyond LPS, other gut-derived mediators may contribute to neuroinflammation. Calprotectin, comprising the S100A8/S100A9 heterodimer, is a marker of intestinal inflammation released mainly by neutrophils, and elevated fecal calprotectin has been reported in a human cohort study of AD [50]. Whether calprotectin mediates gut-to-brain amyloid transmission, and its specificity for gut-mediated neuroinflammation, its ability to predict AD progression, and its value as an AD biomarker remain to be determined.

Trimethylamine *N*-oxide (TMAO) has emerged as a further significant gut-derived factor in AD pathophysiology. Trimethylamine, a byproduct of gut bacterial breakdown of dietary choline, carnitine, and betaine, is converted to TMAO in the liver, and growing evidence points to TMAO as an important signaling molecule at the gut–brain interface. Increased blood TMAO levels have been linked to hippocampal atrophy, increased amyloid-β deposition, and worsened tau pathology. Recent genetic and observational analyses have suggested an association between TMAO-related pathways and AD risk; however, causal interpretation remains limited by potential pleiotropy, instrument validity and population-specific effects. In a senescence-accelerated mouse model, TMAO administration was associated with insulin resistance and cognitive deficits [51]. Mechanistically, TMAO affects the central immune system by altering microglial function; notably, it downregulates P2Y12 receptors in some microglial subpopulations, particularly in the paraventricular nucleus, leading to increased sympathetic activity and stronger neuroinflammatory signaling in regions important for autonomic regulation [52]. These findings support TMAO as a candidate systemic mediator linking gut microbial metabolism with neuroinflammatory and neurodegenerative pathways, but its causal and clinical significance in human AD remains unresolved [51,52].

*The cross-seeding hypothesis*: A further mechanism connecting gut inflammation to AD, important but not widely recognized, involves structural mimicry between bacterial and human amyloids. E. coli and Salmonella, Gram-negative enteric bacteria, produce functional amyloid proteins such as curli fibers as part of their biofilm structure, and these bacterial amyloids show substantial structural similarity to human amyloid-β [53]. When the intestinal barrier is compromised, bacterial amyloids can enter the bloodstream and reach the brain, where they may interact with endogenous Aβ peptides and act as templates that accelerate misfolding and aggregation in a manner resembling prion-like propagation. This raises the possibility that, in certain cases, the initial “seed” for amyloidogenesis in AD may be microbial rather than neuronal in origin [53]. This perspective challenges the conventional neuron-centric view of AD and highlights novel therapeutic avenues focused on limiting bacterial amyloid formation, translocation, or interaction with host proteins.

*Adaptive immunity and T-cell neuroinflammation*: The adaptive immune system also contributes significantly to neuroinflammation in AD, with BBB dysfunction facilitating T-cell infiltration into the brain in a manner that correlates with neuronal loss [54]. Infiltrating T cells, particularly CD8^+^ T cells, dynamically interact with resident microglia, enhancing microglial activation and neurodegeneration; this interaction triggers release of pro-inflammatory mediators such as IFN-γ, TNF-α, and IL-17, activating further neuroinflammatory pathways [55]. The presence of CD8^+^ T cells together with microglia creates a synergistic effect that exacerbates cellular damage beyond what microglia produce alone [56]. Elevated IFN-γ exacerbates neuroinflammation in APP/PS1 mice by activating microglia and astrocytes, leading to Aβ deposition and cognitive decline, and serves as a key AD biomarker, particularly in APOE ε4 carriers, influencing microglial phenotype via ACSL1 expression [57].

In addition to cytokines, chemokines represent another important family of inflammatory mediators in AD neuroinflammation. Chemokines such as CXCL1, CCL2, and CX3CL1 mediate immune cell chemotaxis toward amyloid plaques, where they promote amyloid clearance but can also chronically induce neurotoxicity. Imbalance between pro- and anti-inflammatory signaling promotes neurodegeneration through several mechanisms, including bystander damage to nearby neurons during inflammatory mediator release. Although intended to promote amyloid clearance, overstimulation of chemokine signaling recruits large numbers of immune cells and causes bystander neuronal damage [40].

### 5.3. The Kynurenine Pathway

One of the most important but underrecognized molecular links connecting peripheral immune activation to neurodegeneration in AD is the kynurenine pathway (KP), the major degradation pathway for the essential amino acid tryptophan. Over 90% of cellular tryptophan is metabolized through the KP, with the remainder utilized for serotonin synthesis. Within the KP, tryptophan is converted to kynurenine through two rate-limiting enzymes, tryptophan 2,3-dioxygenase (TDO) and indoleamine 2,3-dioxygenase (IDO). Under normal physiological conditions, the KP has neuroprotective and immunoregulatory functions; however, during inflammation in AD, the KP becomes dramatically dysregulated [21].

IDO is expressed throughout the body at relatively low physiological levels but is transcriptionally upregulated in response to immune activation or chronic stress via its main inflammatory drivers, including interferon-γ (IFN-γ), LPS, TNF-α, and TLR ligands. This produces a corresponding systemic increase in kynurenine both peripherally and within the brain. This is mechanistically relevant to AD given that LPS-TLR4 signaling and IFN-γ production, induced by gut dysbiosis and peripheral T-cell activation, directly induce IDO activity, shunting tryptophan metabolism toward potentially neurotoxic downstream metabolites [58,59].

**Figure 5:**
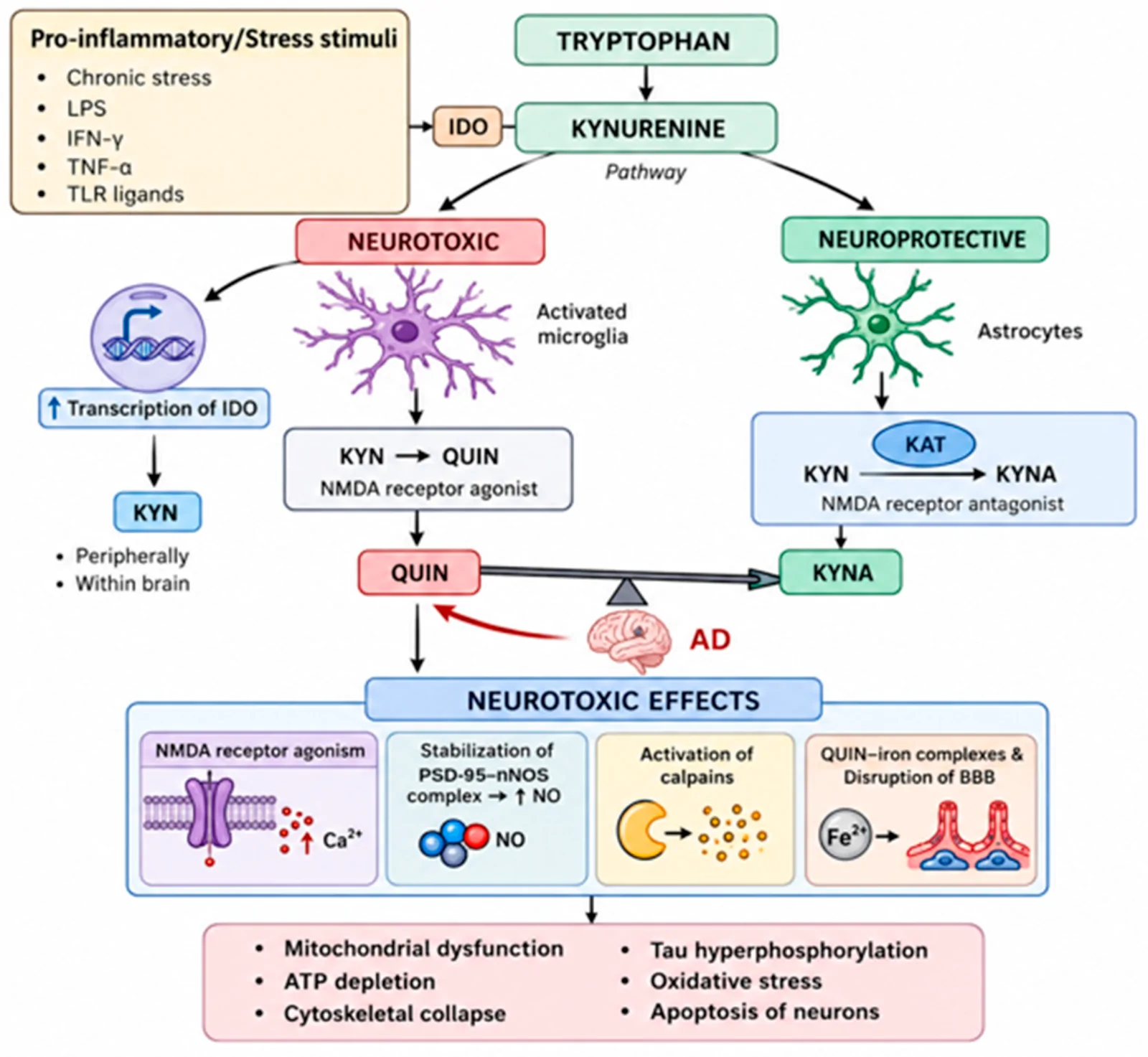
The kynurenine pathway and its neurotoxic-versus-neuroprotective branches. Chronic stress, lipopolysaccharide (LPS), interferon-γ (IFN-γ), TNF-α, and Toll-like receptor (TLR) ligands upregulate indoleamine 2,3-dioxygenase (IDO) and increase peripheral and central kynurenine (KYN). Activated microglia preferentially generate quinolinic acid (QUIN), an NMDA receptor agonist with neurotoxic effects (mitochondrial dysfunction, oxidative stress, tau hyperphosphorylation, and apoptosis), while astrocytes use kynurenine aminotransferase (KAT) to generate the neuroprotective NMDA receptor antagonist kynurenic acid (KYNA).

The cell type responsible for metabolizing kynurenine plays a pivotal role in determining whether the pathway proceeds along a neurotoxic or neuroprotective route. Activated microglia and macrophages preferentially convert kynurenine into quinolinic acid (QUIN), an NMDA receptor agonist. Astrocytes, in contrast, metabolize kynurenine via kynurenine aminotransferases (KATs) into kynurenic acid (KYNA), an NMDA receptor antagonist that is neuroprotective by preventing excitotoxicity [60]. Figure 5 depicts this bifurcation, showing how the same upstream triggers (chronic stress, LPS, IFN-γ, TNF-α, and TLR ligands acting on IDO) drive kynurenine production, while the downstream cell type (microglia versus astrocyte) determines whether the pathway proceeds toward the neurotoxic QUIN/NMDA-agonist branch or the neuroprotective KYNA/NMDA-antagonist branch.

Under pathological conditions where QUIN accumulates in excess, it induces neuronal excitotoxicity and neurodegeneration through several converging mechanisms: high-affinity NMDA receptor agonism, leading to calcium overload; stabilization of the PSD-95–nNOS complex, resulting in excessive nitric oxide and peroxynitrite production; and activation of calpains. Concurrently, QUIN–iron complexes induce reactive oxygen species and lipid peroxidation products, causing NMDA receptor sensitization and disruption of the BBB. These pathways collectively lead to mitochondrial dysfunction, ATP depletion, cytoskeletal collapse, tau hyperphosphorylation, oxidative stress, and neuronal apoptosis [58,60], directly linking the kynurenine pathway to tau pathology. KYNA, by contrast, is neuroprotective and antagonizes the neurotoxic actions of QUIN through several mechanisms: it acts as a non-selective NMDA receptor antagonist, blocks excitatory neurotransmission mediated by AMPA receptors (thereby reversing QUIN-induced excitotoxicity), and acts as a negative allosteric modulator of α7 nicotinic acetylcholine receptors, decreasing presynaptic glutamate release and interrupting the QUIN-induced positive feedback cycle of calcium influx. Loss of this KYNA-mediated regulation in AD therefore leaves neurons more susceptible to QUIN-driven excitotoxicity [58].

### 5.4. Microbial Metabolites and Neurobiological Effects

*Short-chain fatty acids*. SCFAs, mainly acetate, propionate, and butyrate, are microbially generated metabolites resulting from colonic fermentation of dietary fiber and resistant starch, typically containing 2–6 carbon atoms. They are produced primarily by colonic microbes, including Bacillus spp., Bifidobacterium spp., and Clostridium spp., and bind G-protein-coupled receptors to initiate signaling cascades that modulate immune and inflammatory activity, including regulation of cytokines such as TNF-α, IL-1β, and IL-6 in a context-dependent manner [61]. Under physiological conditions, SCFAs are important mediators of intestinal homeostasis, mucosal immune function, and BBB integrity. In AD pathology, gut depletion of these protective metabolites secondary to dysbiosis converts this system from a protective one into one that permits neurodegeneration.

Of the three major SCFAs, butyrate has received the most attention regarding neuroprotective mechanisms, including reduction of pro-inflammatory cytokine secretion through HDAC inhibition. This suppression alleviates microglial hyperactivation and Aβ deposition while improving synaptic plasticity [62]. SCFAs can also cross the blood–brain barrier directly; once in the brain, they exert anti-inflammatory effects that may ameliorate AD symptoms, with reductions in hippocampal pro-inflammatory molecules such as IL-6 observed following SCFA exposure [61]. Propionate, generated by gut microbes fermenting dietary fibers such as β-glucan, has been shown to affect immune cells and regulate intestinal inflammation, as well as barrier integrity. Interestingly, recent studies have shown that while propionate reduces microglial activation, it also inhibits microglial phagocytic activity, highlighting seemingly contradictory effects on microglia; this matters because therapies that increase SCFAs non-specifically may produce opposing and competing effects in the brain [61]. SCFAs secreted by gut microbes appear to regulate microglial function in the CNS through at least two pathways: a central pathway in which SCFAs access brain parenchyma through the circulation and directly affect microglia, and a peripheral pathway in which SCFAs act on receptors expressed by peripheral immune cells (neutrophils and monocytes) to induce cytokine production and trafficking of immune cells into the brain, indirectly affecting microglia [63].

*Tryptophan metabolites*. Dietary tryptophan is metabolized by gut microorganisms via three major pathways: the kynurenine pathway, the indole pathway, and the serotonin pathway. Each produces neuroactive metabolites with largely opposing neurobiological effects, and dysregulation of these pathways in AD links microbial dysbiosis to neuroinflammation in the CNS. Important microbiota-derived tryptophan metabolites include indole derivatives such as indole-3-pyruvic acid, indole-3-acetaldehyde, and indoxyl sulfate. The kynurenine pathway is activated by indoleamine and tryptophan 2,3-dioxygenases (IDOs/TDOs) in intestinal host cells and produces kynurenine metabolites that are powerful agonists of aryl hydrocarbon receptor (AhR) signaling, an evolutionarily conserved, ligand-activated transcription factor that also modulates developmental biology and tissue senescence [64].

Under homeostatic conditions, microbial metabolism of tryptophan into indole metabolites represents an important dietary reservoir of neuroprotective AhR ligands. Gut-derived tryptophan metabolites such as indole, generated via bacterial tryptophanase, modulate astrocytes and microglia through AhR signaling pathways that play crucial roles in neuroinflammation. Indole derivatives activate AhR signaling, inhibit the NF-κB pathway, and decrease formation of NLRP3 inflammasomes. Oral administration of indole, indole-3-acetic acid (IAA), and indole-3-propionic acid (IPA) to tryptophan-free diet-fed transgenic APP/PS1 mice for four weeks ameliorated AD associated neuropathology through prominent suppression of inflammatory markers, upregulation of AhR, inhibition of the NF-κB pathway, and downregulation of NLRP3 inflammasome formation, leading to decreased levels of TNF-α, IL-6, IL-1β, and IL-18 [64].

Serotonin (5-hydroxytryptamine; 5-HT) biosynthesis begins with the rate-limiting enzyme tryptophan hydroxylase 1 (TPH1), which converts L-tryptophan to 5-hydroxytryptophan; decarboxylation of 5-hydroxytryptophan yields serotonin [65]. The gut microbiota can indirectly modulate central serotonergic pathways through regulation of tryptophan and tryptamine availability, suggesting a bidirectional microbiota–serotonin axis whereby microbial dysbiosis in AD drives depletion of the serotonin precursor pool while simultaneously diverting tryptophan metabolism toward neurotoxic kynurenine pathway derivatives rather than serotonin and neuroprotective indole derivatives [66].

Lipopolysaccharides and bacterial amyloids: In addition to fermentation byproducts and amino acid breakdown products, intestinal dysbiosis contributes to two structurally distinct but mechanistically synergistic danger signals that interact with and exacerbate.

### 5.5. AD Pathology: LPS and Bacterial Amyloids

LPS is an integral component of the outer membrane of Gram-negative bacteria and is among the most powerful stimulators of innate immune activation. Several studies have implicated increased levels of Bacteroides fragilis and Escherichia coli in AD patients due to their capacity to elevate serum LPS concentrations and contribute to cognitive impairment [67]. Numerous studies have documented elevated bacterial LPS in AD brain lysates relative to controls, including accumulation of LPS in neocortical and hippocampal neurons and co-localization of LPS with Aβ in amyloid plaques and perivascular Aβ deposits. This co-localization within the brain positions LPS as a contributor to plaque biology rather than merely a systemic initiator [68,69,70].

In addition to LPS, intestinal bacteria themselves generate functional amyloid proteins that are structurally and immunologically homologous to human amyloidogenic proteins. Functional amyloid proteins secreted by several bacterial genera, including Streptomyces, Staphylococcus, Bacillus, and Escherichia, are structurally and immunologically homologous to the human Aβ42 peptide and may serve as pathogen-associated molecular pattern recognition molecules. Amyloids produced by bacteria may contribute to AD pathology by binding microglial TLR2, leading to microglial activation via upregulation of Notch1 signaling [71,72]. The most studied bacterial amyloid is curli, secreted by most E. coli strains. Curli has been shown to directly bind human amyloids and may enhance deposition and aggregation of amyloid fibrils; preformed fibrils of the CsgA and CsgB curli subunit proteins could seed Aβ amyloid formation by reducing the lag phase in a dose-dependent manner, thus enhancing neurodegeneration [71].

Bacterial amyloids also appear to act through a neuroanatomical propagation pathway: functional bacterial amyloids can promote misfolding of human amyloids, and disease-causing aggregation may begin in the enteric nervous system and propagate through the vagus nerve to the brain. Bacterial amyloids additionally provoke inflammation within their host, and activation of inflammatory pathways and neuroinflammation by bacterial amyloids can promote disease progression through chronic inflammation [71]. LPS and bacterial amyloids have each been shown, in experimental systems, to influence innate immune activation or amyloid fibrillization. Whether these effects occur at physiologically relevant concentrations and contribute meaningfully to human AD remains uncertain. Although LPS and bacterial amyloid-related signals have been detected in association with AD-related findings, current evidence is insufficient to conclude that either is essential for gut-to-brain transmission of amyloid pathology.

## 6. Gut–Brain Axis and Amyloid-Beta Pathology

The mechanistic pathways reviewed above, intestinal barrier dysfunction, endotoxemia, the kynurenine pathway, and microbial metabolite signaling, converge most directly on amyloid-beta (Aβ) biology. This section examines that convergence in detail: how gut microbiota influences Aβ production, aggregation, and clearance, and how this evidence has begun to translate from preclinical models into human observational and interventional studies. Much of the mechanistic evidence in this section derives from transgenic mouse models. While these models provide instrumental insights, they do not fully recapitulate sporadic, late-onset human AD. Human data currently support association rather than causation.

### 6.1. Influence on Amyloid Production and Aggregation

Alterations of the gut microbiota have also been shown to affect amyloid-beta pathology independently of neuroinflammation. They can modulate both direct and indirect pathways involved in the biochemical generation of Aβ, as well as the morphological processes responsible for its subsequent polymerization into cytotoxic oligomers and insoluble plaques. Emerging evidence suggests a role for the gut microbiome in upstream regulation of amyloidogenesis itself.

Gut-derived Aβ contribution to plaque burden: One of the most intuitively interesting yet often overlooked aspects of this relationship is that the gut produces Aβ directly. Aβ originating outside the CNS, including that found in the gut, may contribute to brain Aβ plaque deposition; elevated Aβ levels have been found along the entire gut in both mice and humans. Gut Aβ42 levels increase with age in both wild-type and mutant APP/PS1 mice. This peripheral pool of Aβ, regulated by gut microbes and age-related dysbiosis, could provide an additional amyloidogenic source that supplements CNS-produced Aβ and accelerates plaque deposition beyond what would be expected from CNS production alone [73].

Microbial modulation of APP-cleaving enzymes: Gut dysbiosis also regulates APP-cleavage enzymes upstream of Aβ production. Aβ is generated through hydrolysis of amyloid precursor protein (APP) via the amyloidogenic pathway, in which beta-site APP cleaving enzyme 1 (BACE1) initiates Aβ production by cleaving APP into a large soluble protein and a 99-amino-acid *C*-terminal fragment (C99); subsequent cleavage of C99 by γ-secretase generates Aβ in either its 40- or 42-amino-acid form, with Aβ42 considered particularly significant in early AD. Gut dysbiosis can enhance production through this pathway at several stages: pro-inflammatory cytokines arising from dysbiosis can promote AD pathology by upregulating expression of β-APP and BACE1 activity, and microbial metabolites can influence γ-secretase activity. Owing to structural similarities between microbial metabolites and amyloid proteins, these metabolites may also induce aberrant protein folding through a prion-like mechanism [74]. As outlined in Section 5, many of these effects are thought to be mediated, in part, through activation of the LPS–TLR4–NF-κB–microglial cascade, which promotes neuroinflammatory signaling and may secondarily influence APP processing.

A further layer of regulation involves gut-derived bile acids. Bile acids synthesized by gut microbiota have also been associated with elevated Aβ production in AD. Notably, bile acids may weaken BBB integrity by inhibiting tight junctions between BBB endothelial cells, increasing the influx of bile acids, along with cholesterol, into the CNS from the periphery. Increased cholesterol influx leads to increased cholesterol binding to APP within the brain, facilitating transport of APP into lipid rafts and increasing β-secretase-mediated cleavage of APP with consequent Aβ production. Bile acids may additionally impair the brain’s capacity to clear cholesterol [75,76,77].

Cross-seeding mechanisms: In addition to increasing Aβ production, microbial products directly promote faster Aβ fibrillation through cross-seeding interactions. Models established both in vivo and in vitro demonstrate that microbial amyloids produced in the gut may promote Aβ cross-seeding and prime the innate immune system to respond more strongly and pathologically to brain amyloids, potentially driving age-related neurodegeneration. Curli, the amyloid protein produced by E. coli, has been the most studied molecule in this process [71,74].

Antibiotic-induced dysbiosis further supporting evidence: In a study by Tettevi and colleagues, treatment with broad-spectrum antibiotics caused dramatic gut dysbiosis (decreases in Firmicutes and Bacteroidetes of more than 90%) in 3xTg-AD mice. This dysbiosis produced deficits in spatial working memory and anxiety-like behavior, as well as decreased locomotor activity. The mice showed region-specific dysregulation of cholinergic genes, significantly increased hippocampal and cortical TNF-α expression, and increased brain amyloid-β1–42 and tau levels. These data show that disruption of the gut microbiome can potentiate late-stage AD pathology by promoting cognitive deficits, neuroinflammation, and aggregation of key proteins [78].

### 6.2. Microbiota-Driven Modulation of Amyloid Clearance

Microglia are considered the principal mediators of Aβ clearance in the brain, and gut microbial signals directly modulate microglial phagocytic capacity. Germ-free AppNL-G-F AD mice presented with decreased amyloid-β pathology, along with a shift of microglial cells toward a less inflammatory phenotype with heightened phagocytic activity. Notably, bacterial extracellular vesicles obtained from fecal slurry of specific-pathogen AD mice could nullify these germ-free effects on microglial engagement and plaque deposition, providing the first evidence that bacterial extracellular vesicles derived from commensal gut microbiota substantially influence AD pathology [79,80].

Specific bacterial species, particularly Bacteroides fragilis, have emerged as significant suppressors of microglial clearance. Bacteroides fragilis suppresses microglial phagocytic ability, resulting in Aβ clearance defects and amyloid plaque deposition; administration of metronidazole to deplete gut Bacteroidota in aged 5xFAD mice increased microglial expression of genes involved in phagocytosis, cytokine signaling, and lysosomal degradation. These findings suggest that members of the Bacteroidota phylum, including Bacteroides fragilis, may suppress microglial amyloid clearance mechanisms in AD [81]. Bacteroides fragilis and its metabolites, 12-hydroxyheptadecatrienoic acid (12-HHTrE) and prostaglandin E2 (PGE2), also induce microglial activation and upregulate the pro-inflammatory cytokines IL-1β and IL-6, worsening neuroinflammation and contributing to Aβ plaque deposition and tau phosphorylation, with consequent cognitive impairment [67]. As described in Section 5, these effects are likely reinforced by LPS–TLR4–NF-κB–driven neuroinflammatory signaling, although direct evidence in humans is limited [67].

One manifestation of gut dysbiosis observed in AD is depletion of SCFAs, especially butyrate, which are critical for maintaining homeostatic microglial phenotypes. This depletion intensifies microglial hyperactivation while simultaneously impairing Aβ phagocytosis, yielding microglia with both heightened reactivity and diminished amyloid clearance capacity [82].

Further support for the idea that restoring gut microbial balance restores amyloid clearance comes directly from fecal microbiota transplantation (FMT) studies. FMT restored gut homeostasis in 5xFAD mice, leading to reduced Aβ pathology and LPS in colon and hippocampus, and suppressed expression of APP, BACE1, and presenilin-1, potentially through inhibition of TLR4/IKKβ/NF-κB signaling. Mechanistically, preclinical FMT studies have found that treatment reduces Aβ plaques, normalizes tau phosphorylation, suppresses inflammasome activation, and restores microglial homeostasis by modulating microbial metabolites and immune signaling pathways [83].

While these preclinical findings are mechanistically compelling, human evidence linking specific taxa or microbial products to Aβ clearance remains indirect and largely associative.

### 6.3. Clinical Evidence in Human Studies

A growing body of evidence supports translation of these experimental results to human populations. In one study, treatment outcomes were assessed in a small sample of ten dementia patients with severe Clostridioides difficile infection (CDI) treated with FMT, compared with ten control dementia patients with CDI treated with antibiotics. Patients were assessed with the Mini-Mental State Examination (MMSE) and Clinical Dementia Rating Sum of Boxes (CDR-SB) one month before and after treatment. FMT-treated patients showed clinical and cognitive improvement on both measures relative to controls: MMSE scores improved to a median of 16.00 (IQR 13.00–18.00) after FMT compared with 10.0 (IQR 9.8–15.3) after antibiotics, while CDR-SB scores improved to a median of 5.50 (IQR 4.00–8.00) after FMT compared with 8.0 (IQR 7.9–12.5) after antibiotics. FMT treatment also produced overall differences in gut microbial composition, with enrichment of Proteobacteria and Bacteroidetes, and patients who underwent FMT showed significant differences in metabolic pathways related to alanine, aspartate, and glutamate metabolism [84].

A second study of five Alzheimer’s dementia patients similarly observed significant cognitive improvement after FMT: MMSE scores improved from a median of 13.00 before treatment to 18.00 afterward, Montreal Cognitive Assessment (MoCA) scores increased from 12.0 at baseline to 21.0 after three months, and CDR-SB scores decreased from 10.00 to 5.50. Microbiome analysis showed enrichment in Bacteroidaceae and depletion in Enterococcaceae among patients who underwent FMT, and gene expression profiling revealed differences in lipid metabolism genes. Together, these results point to gut microbial restoration through FMT as a potential route to improved cognition via alterations in gut microbiota, metabolic function, and host gene expression [85].

Supporting these interventional findings, human observational studies have begun to corroborate the gut–brain-axis hypothesis by revealing links between intestinal inflammation and central aspects of AD pathology. One limitation of earlier human studies was the sole assessment of microbiome composition without quantification of intestinal inflammation itself. Addressing this gap, one notable human cohort study measured intestinal inflammation directly through fecal calprotectin, a validated marker of intestinal inflammation and barrier dysfunction, in older adults spanning the AD spectrum, relating it to clinical diagnosis, age, CSF biomarkers, ^11^C-Pittsburgh compound B PET (PiB-PET) amyloid burden, and cognitive function. Fecal calprotectin levels increased with age and were significantly higher in amyloid-confirmed AD dementia patients, in whom higher calprotectin levels were associated with higher PiB-PET amyloid burden; calprotectin levels also correlated with CSF markers of AD pathology and declining verbal memory scores among cognitively healthy individuals with preclinical amyloid deposition. These clinical observations further implicate intestinal inflammation in AD pathogenesis [50].

A randomized double-blind crossover pilot trial demonstrated that dietary interventions known to modulate the gut microbiome can produce changes in CSF biomarkers reflective of AD pathology. Seventeen patients (11 with mild cognitive impairment and six cognitively normal controls) followed either six weeks of a modified Mediterranean–ketogenic diet (MMKD) or six weeks of an American Heart Association diet (AHAD), separated by a four-week washout period, with gut microbiome composition, plasma SCFAs, and CSF AD markers measured before and after each intervention. Each diet altered gut microbiome composition and SCFA production in distinct ways, some of which correlated with CSF Aβ-42 levels, indicating that patients with MCI harbor distinct fungal–bacterial networks that respond differently to dietary intervention; the MMKD appeared to promote greater microbiome remodeling in MCI subjects compared with cognitively normal participants [86].

Building on these observations, large-scale cross-sectional human studies have correlated compositional shifts in the microbiome with worsening cognitive impairment and AD-associated biomarker alterations. In one of the largest cross-sectional human studies examining gut microbiota across the cognitive spectrum to date, fecal microbiota was analyzed from 94 cognitively normal controls, 125 individuals with mild cognitive impairment, and 83 AD dementia patients from a large Chinese cohort, using 16S rRNA sequencing to identify stage-specific microbial changes. Microbial compositional changes were shown to be stage-dependent across the cohort, and mediation analysis implicated immune, BBB, and metabolic biomarkers as messengers in microbiota–gut–brain-axis signaling, identifying stage-associated taxa correlated with established CSF AD biomarkers, such as amyloid-β and phosphorylated tau [87].

While these studies establish strong correlations between gut dysfunction and AD pathology through dietary and observational analyses, causation cannot be determined from such designs alone. To address this, a recent large-scale population-based study used Mendelian randomization analysis to estimate causal effects between risk factors and AD, leveraging genetic variants as instrumental variables to approximate a natural experiment. Mendelian randomization and colocalization analysis of 629 gut microbiota phenotypes and 2103 immune, BBB, and metabolic biomarkers identified putative causal relationships between mucin-degrading bacteria, SCFA-producing microbes, and PD-1/PD-L1-related biomarkers and decreased AD risk, while cardiovascular-associated microbes, Aβ-related proteins, and lipoproteins were estimated to increase AD risk. This study provides genetically anchored support for a possible contribution of the gut microbiome to AD risk [88]. However, Mendelian randomization estimates rest on assumptions that cannot be fully verified: horizontal pleiotropy may violate the exclusion restriction; genetic instruments for microbial taxa are often few and weak; and most instruments derive from largely European-ancestry cohorts, thus limiting generalizability. The findings are therefore compatible with, but do not prove, a causal role, and they are consistent with a bidirectional model.

Mediation analyses from this study further suggested a role for CD28^−^CD8^+^ T cells, CD19 on IgD^+^CD24^+^ B cells, glycoproteins, and low-density lipoprotein in mediating microbiota–gut–brain-axis communication, while colocalization analyses provided further support for shared genetic contributions to AD and lipid metabolism via the variant rs7412 [88]. Taken together, these findings are compatible with a bidirectional model of gut–brain feedback in AD, in which gut dysbiosis may contribute to neuroinflammation, immune ageing, blood–brain barrier dysfunction, and SCFA dysregulation, while AD pathology may in turn influence the gut microbiota via altered lipid metabolic processes; in humans, this model remains hypothetical. Having considered the evidence for amyloid-beta, we now turn to parallel evidence implicating the gut–brain axis in tau pathology.

## 7. Gut–Brain Axis and Tau Pathology

The previous section traced how gut dysbiosis and microbial signaling converge on amyloid-beta biology. Tau pathology, the second defining hallmark of AD, is influenced by an overlapping but mechanistically distinct set of gut-derived processes, centered on inflammation-driven kinase activation and oxidative or mitochondrial dysfunction.

### 7.1. Inflammation-Mediated Tau Phosphorylation

Although neuroinflammatory processes driven by activation of microglia and astrocytes were once seen as effects of amyloid-beta buildup, recent research suggests that they are key drivers of cognitive decline rather than merely side effects of disease progression. This shift in understanding accompanies a growing appreciation of the gut–brain connection: the microbial communities of the gut substantially influence central inflammation through the microbiota–gut–brain axis [40]. In AD, key short-chain fatty acid (SCFA)-producing bacteria are significantly reduced. SCFAs are essential for host nutrition and colonic epithelial health, while also regulating mucosal barrier permeability; they further exert protective effects on the blood–brain barrier and support the maturation and immune function of microglia.

Multiple human clinical investigations have identified reduced levels of beneficial gut bacteria, including Bifidobacterium and Lactobacillus, alongside increased levels of harmful bacteria such as Escherichia and Streptococcus, in individuals with AD compared with healthy individuals [89]. In rodents and humans, particular gut microbiota taxa, including Bacteroides vulgatus, Bacteroides distasonis, Lactobacillus salivarius, Clostridium cluster species, and Bifidobacterium longum, may influence neuroinflammatory signaling and brain function [90]. Collectively, these observations imply a possible connection between gut microbiota dysbiosis and the onset and progression of AD.

Gut dysbiosis increases intestinal permeability (“leaky gut”), allowing LPS to enter the bloodstream and drive the TLR4–NF-κB–cytokine cascade described in Section 5, maintaining an inflammatory environment in the brain [91]. Patients with cognitive impairment have also been found to have elevated levels of pro-inflammatory cytokines and lower levels of anti-inflammatory cytokines, such as IL-10, in peripheral blood, with pro-inflammatory cytokine levels positively correlated with the abundance of Escherichia/Shigella [9].

A cross-sectional multi-omics study by Ferreiro and colleagues showed that people with high cerebrospinal fluid (CSF) tau biomarkers had distinctive gut microbial profiles, rich in pro-inflammatory Proteobacteria and low in Bacteroidetes, further supporting the link between neuroinflammation and tau [15]. The activation state of microglia also appears to be partly governed by gut-derived signals: TREM2 (triggering receptor expressed on myeloid cells 2), an important microglial receptor that influences inflammatory responses, shows altered expression in the context of gut dysbiosis-driven inflammation. These processes are consistent with the LPS–TLR4–NF-κB–microglial cascade outlined in Section 5; however, the relative contribution of gut-derived versus brain-intrinsic drivers of tau pathology in humans remains unclear.

### 7.2. Oxidative Stress and Mitochondrial Dysfunction

In AD, oxidative stress and mitochondrial dysfunction are tightly interconnected in a self-amplifying cycle that drives disease progression. Early mitochondrial impairment, particularly in hippocampal and cortical neurons, leads to deficits in oxidative phosphorylation and ATP production, often preceding hallmark pathologies such as amyloid-beta plaques and tau tangles. Dysfunctional mitochondria generate excessive mitochondrial reactive oxygen species (mtROS), a key contributor to oxidative stress that damages mitochondrial DNA, proteins, and lipids [92].

As outlined in Section 5, LPS-induced activation of the TLR4–NF-κB–microglial cascade promotes oxidative stress via NADPH oxidase (NOX)-derived superoxide and peroxynitrite formation, which can nitrosylate and oxidize tau protein, promoting its abnormal phosphorylation and oligomerization [93,94]. Mitochondrial dysfunction plays a central role in this oxidative process: tau hyperphosphorylation disrupts mitochondrial dynamics by interfering with the interaction between tau and the outer mitochondrial membrane, leading to fragmentation and reduced electron transport chain activity, which further increases ROS production. Gut-derived uremic toxins such as indoxyl sulfate and *p*-cresol sulfate have been proposed to impair mitochondrial function in neuronal cells, but their accumulation in AD and their contribution to AD pathology have not been established.

Emerging research has further demonstrated that mitochondria-associated membranes (MAMs), specialized contact sites between the endoplasmic reticulum and mitochondria that regulate calcium signaling and lipid metabolism, are disrupted in AD [95]; whether they respond to gut-derived inflammatory signals has not been established. MAM dysfunction promotes mitochondrial calcium overload, exacerbates oxidative damage, and creates a permissive environment for tau aggregation. A microbiome–MAM–tau axis is conceivable but remains to be investigated.

Taken together, the evidence establishes a compelling framework in which gut dysbiosis-induced oxidative stress and mitochondrial dysfunction converge on tau biology through multiple complementary mechanisms in preclinical models. Translation of these findings to sporadic human AD requires longitudinal studies integrating microbiome, metabolic, and tau biomarkers.

## 8. Gut–Brain Axis, Neurotransmission, and Synaptic Function

Beyond its effects on amyloid, tau, and neuroinflammation, the gut microbiome also directly shapes the neurotransmitter systems and synaptic mechanisms that underlie cognition, and these effects manifest behaviorally in ways that are increasingly well documented in both animal and human studies. This section addresses those three layers in turn.

### 8.1. Neurotransmitter Modulation

The gut microbiome is a key endogenous source of neuroactive chemicals and exerts regulatory control over numerous classical neurotransmitter systems implicated in cognition. In the gut, specialized endocrine cells known as enterochromaffin cells, mucosal mast cells, and myenteric neurons produce more than 90% of the body’s serotonin (5-hydroxytryptamine, 5-HT). Gut microbiotas appear to play a role in controlling blood 5-HT levels; mice raised without microbial colonization have much lower serum 5-HT concentrations [96]. Specific gut bacteria, notably spore-forming Clostridia, stimulate enterochromaffin cells to generate 5-HT by upregulating tryptophan hydroxylase 1 (TPH1) [96]. Serotonin in the gut governs gastrointestinal motility and enterocyte function; however, its systemic availability also influences brain serotonergic tone indirectly through tryptophan availability across the blood–brain barrier [7].

Tryptophan is metabolized via three major pathways, of which more than 90% of dietary tryptophan passes through the kynurenine pathway, generating several active metabolites, including kynurenine, kynurenic acid, 3-hydroxykynurenine, 3-hydroxyanthranilic acid, and quinolinic acid. Dysregulation of the kynurenine pathway has been implicated in several conditions, including neurodegenerative and inflammatory neurological diseases, with quinolinic acid playing a key role in their pathogenesis [97,98]. In transgenic models, gut-driven shifts in tryptophan metabolism influence neuroinflammation and behavior; in humans, associations between kynurenine metabolites and AD are emerging but not yet definitive.

Lactobacillus and Bifidobacterium species in the gut produce the primary inhibitory neurotransmitter, gamma-aminobutyric acid (GABA). Decreases in these taxa during dysbiosis lead to insufficient GABAergic tone, increasing neuronal hyperexcitability and disrupting the excitatory/inhibitory balance essential for hippocampal memory encoding. Studies in mice show that Lactobacillus rhamnosus JB-1 alters central GABA receptor expression and reduces anxiety- and depression-related behavior, and that vagotomy abolishes these effects, implicating vagal nerve transmission as a relay channel [99].

Gut microbial regulation also affects the cholinergic system, which is severely impaired in AD. The availability of acetylcholine precursors is partially dependent on fermentation of choline-containing food substrates by gut microbes; however, acetylcholine production pathways compete with choline metabolism by some gut bacteria toward TMAO. Gut-produced neurotransmitter precursors, including dopamine precursors, may also be altered in AD-associated dysbiosis, with possible consequences for dopaminergic reward and attention circuits; however, direct evidence in AD is lacking.

While these mechanisms are well supported in animal models, direct evidence linking specific microbial taxa or metabolites to neurotransmitter alterations in human AD remains limited.

### 8.2. Synaptic Plasticity and Memory Impairment

The hippocampus, comprising the cornu ammonis (CA) regions 1–3, the dentate gyrus, and the subiculum, is the center of learning and memory, and changes in neurochemical signaling underline its structural and functional plasticity [100]. Synaptic plasticity, including long-term potentiation (LTP) and long-term depression (LTD), forms the biological basis of learning and memory, and its disturbance is a crucial early stage in AD pathophysiology. The gut–brain axis regulates synaptic plasticity through several convergent pathways involving metabolite signaling, neurotrophic factors, and inflammatory mediators [101].

One of the most significant regulators of synaptic plasticity is brain-derived neurotrophic factor (BDNF), which stimulates LTP induction, dendritic spine density, and synaptogenesis via TrkB receptor signaling. Reduced hippocampal BDNF expression is strongly linked to gut dysbiosis. Studies in germ-free mice have shown significantly lower hippocampal BDNF levels than in conventionally colonized animals, linked to impaired LTP and deficits in spatial learning tasks. Restoration of SCFA-producing microbiota, especially butyrate-producing Firmicutes, rescues BDNF expression by encouraging histone acetylation at the BDNF gene promoter, an intriguing epigenetic mechanism connecting gut microbiota to synaptic plasticity [62,102].

In the presence of accumulated Aβ, pro-inflammatory cytokines, such as TNF-α and IL-1β, generated by microglia can increase AMPA receptor expression on the postsynaptic membrane, allowing increased ion influx and depolarization. In the early stages of AD, these synaptic alterations together result in enhanced LTP and neuronal hyperactivation in the hippocampus [103]. These cytokine effects are consistent with the LPS–TLR4–NF-κB–microglial cascade described in Section 5; however, the extent to which gut-derived signals drive these changes in human AD is not yet known.

The hypothalamic–pituitary–adrenal (HPA) axis represents a further route by which the microbiome affects synaptic plasticity. By disrupting the gut–HPA feedback loop, dysbiosis encourages cortisol hypersecretion; persistently high cortisol reduces hippocampal LTP by downregulating the NMDA receptor subunit GluN2B via the glucocorticoid receptor, lowering the effectiveness of synaptic transmission. Simultaneously, prolonged HPA activity exacerbates synaptic impairment by promoting neuroinflammation and suppressing hippocampal neurogenesis [104].

Multiple rodent models have associated gut dysbiosis-driven neuroinflammation with loss of the postsynaptic density proteins PSD-95, Shank, and Homer, scaffolding proteins essential for maintaining dendritic spine structural integrity. Expression of these proteins, regulated partly by BDNF-TrkB-PI3K-mTOR signaling, declines progressively in AD, and their preservation has been associated with better cognitive trajectories.

### 8.3. Behavioral and Cognitive Outcomes

In both animal and clinical studies, gut microbiome composition has been increasingly linked to behavioral and cognitive changes in AD, including episodic memory loss, executive dysfunction, mood disorders, and neuropsychiatric symptoms, highlighting the gut–brain axis as a critical determinant of disease trajectory and symptom severity. Growing evidence suggests that the host and its gut microbiota interact symbiotically to control the systemic availability of micronutrients, trophic factors, and neurotransmitters that affect cognitive and behavioral function; conversely, microbiota imbalance may lead to neuroinflammation, functional impairment, and aggravation of disease-specific pathogenic processes, as observed in psychiatric and neurodegenerative disorders [90].

Consistent and reproducible cognitive abnormalities are produced in rodent models of AD when the gut microbiota is manipulated using antibiotics, germ-free environments, or FMT. Compared with conventionally housed counterparts, germ-free APP/PS1 mice show much less amyloid pathology and better performance on the Morris water maze and novel object recognition tests. Conversely, transplanting gut microbiota from aged or AD-model donor mice into young, germ-free recipients increases neuroinflammatory markers and reproduces cognitive impairments, demonstrating that gut microbial communities alone can transfer cognitive traits between hosts [105].

Cross-sectional analyses in human studies are consistent with the broader theory that changes in gut microbiota are associated with AD development; however, directionality and causality cannot be inferred from these designs. The association between microbial diversity indices and Mini-Mental State Examination (MMSE) scores highlights a possible role for gut microbiota in preserving cognitive health: reduced diversity may indicate a less resilient gut ecology, more vulnerable to external shocks, inflammation, and metabolic dysfunction, all of which have been linked to neurodegeneration [106]. However, taxonomic patterns differ across cohorts, and no consistent, generalizable microbiome signature of AD has yet been established.

Neuropsychiatric manifestations of AD, including depression, anxiety, agitation, and apathy, are also significantly modulated by gut–brain-axis dysfunction. The microbiota–gut–brain concept has been extended to these behavioral phenotypes, with evidence implicating serotonergic, GABAergic, and HPA-axis dysregulation downstream of gut dysbiosis. Probiotic clinical trials in AD patients have shown improvements in anxiety and depression ratings in addition to MMSE scores, further supporting gut microbial modulation as a practical approach to comprehensive AD symptom management. In a randomized, double-blind, controlled trial in patients with AD, combined probiotic and selenium supplementation improved cognitive and metabolic outcomes [107]; probiotics may also influence production of neurotransmitters such as GABA, norepinephrine, serotonin, dopamine, and acetylcholine via the gut–brain axis, and selenium may lower pro-oxidant and raise antioxidant levels in the brain; however, these mechanisms are inferred rather than demonstrated in these patients. These findings suggest that microbiome-targeted strategies may affect both cognitive and neuropsychiatric dimensions of AD, although larger, longer human trials are needed.

## 9. Targeting the Gut–Brain Axis for Therapeutic Intervention in AD

Given the increasing evidence for the role of the gut–brain axis in AD pathogenesis, there is great interest in targeting this axis as a supplemental or adjunctive therapeutic approach. Strategies include dietary modification, supplementation with live microorganisms, fecal microbiota transplantation (FMT), and innovative pharmacological approaches. This section provides a critical overview of each class of intervention, summarizing the mechanistic basis, preclinical data, and growing clinical evidence.

Nutrition-led translational narrative: Given the central role of diet in shaping gut microbiota composition and function, nutritional strategies represent the most immediately translatable avenue for modulating the microbiota–gut–brain axis in AD. This section prioritizes evidence for dietary patterns (e.g., Mediterranean, MIND, plant-forward, and high-fiber diets), prebiotics, probiotics, and synbiotics, emphasizing their effects on microbial composition, barrier integrity, immune signaling, and cognitive outcomes. Pharmacological approaches that intersect with gut-derived pathways (e.g., GLP-1 receptor agonists) are discussed subsequently as complementary strategies that build on mechanistic insights from nutritional and microbiome-targeted interventions.

### 9.1. Dietary Interventions

Mediterranean and MIND diets: Diet is one of the most potent modulators of gut microbiota composition and diversity. The Mediterranean diet, characterized by high consumption of vegetables, fruits, legumes, whole grains, olive oil, and seafood, and moderate red wine intake, has been consistently linked with lower risk of cognitive decline and dementia in prospective epidemiological studies [108,109]. A landmark cohort study of 923 older adults found that adherence to the Mediterranean–DASH Intervention for Neurodegenerative Delay (MIND) diet, a hybrid of the Mediterranean and Dietary Approaches to Stop Hypertension (DASH) diets, was associated with a 53% reduction in AD risk in the highest adherence tertile compared with the lowest, after adjustment for confounders like age, sex, education, and comorbidities [110]. A systematic review specifically evaluating Mediterranean diet adherence, gut microbiota composition, and neurodegenerative disease risk confirmed that higher adherence was associated with a lower risk of both Alzheimer’s and Parkinson’s disease, identifying several bacterial taxa that were consistently altered in the same direction across both diseases [111]. Direct interventional support comes from a randomized trial in midlife adults at increased genetic risk of AD, in which twelve months of Mediterranean-diet counselling produced more favorable AD biomarker and cerebral perfusion profiles than a typical Western diet, strengthening the case that the observational associations described above reflect a modifiable, diet-driven relationship rather than reverse causation alone [112].

Mechanistically, the Mediterranean diet induces enrichment of SCFA-producing bacteria such as Faecalibacterium prausnitzii, Roseburia intestinalis, and Akkermansia muciniphila, while inhibiting pro-inflammatory taxa such as Proteobacteria and certain Enterobacteriaceae [113]. In the one-year NU-AGE randomized trial in 612 older adults across five European countries, a Mediterranean-diet intervention altered gut microbiome composition, with changes in taxa linked to short-chain fatty acid production, reduced frailty, and improved cognitive function [113]. Observational data from an Australian ageing cohort also link dietary patterns to cognitive decline [114].

Plant-based meals contain polyphenols, including resveratrol, quercetin, curcumin, and epigallocatechin-3-gallate (EGCG), that are poorly absorbed in the upper gastrointestinal tract and reach the colon, where they are extensively metabolized by the microbiota into bioactive compounds. These metabolites exert antioxidant and anti-inflammatory activity, inhibit BACE1 activity, and diminish Aβ aggregation in vitro and in transgenic AD mouse models [115].

Prebiotic substrates and dietary fiber: Commensal saccharolytic bacteria mostly use dietary fiber as their nutritional substrate. The prebiotic effect refers to selective stimulation of growth and metabolic activity of health-promoting bacteria by fermentable fibers such as inulin, fructooligosaccharides (FOSs), galactooligosaccharides (GOSs), and resistant starch [116]. Short-chain fatty acids produced by fermentation of such fibers are thought to mediate many of their effects on gut–brain communication [117].

In animal experiments, high-fiber diets reduced amyloid plaque load and hippocampal microglial activation while increasing BDNF expression and Morris water maze performance in 5xFAD mice; these effects were eliminated by concurrent antibiotic treatment [118]. This dependence on an intact microbiota supports—but, based on a single rodent study, cannot, on its own, confirm—a role for the gut microbiome in this phenotype. The ketogenic diet (KD), very low in carbohydrate and high in fat, is a further dietary pattern of relevance to AD. KD increases circulating ketone bodies (beta-hydroxybutyrate and acetoacetate), which provide alternate neuronal fuel in the context of impaired cerebral glucose metabolism, a well-established feature of AD that has itself been targeted directly in small clinical trials of ketogenic agents in mild-to-moderate AD [119]; KD also inhibits the NLRP3 inflammasome, a critical driver of neuroinflammation, and preferentially shifts the microbiota toward increased abundance of Akkermansia [120,121].

### 9.2. Probiotics, Prebiotics, and Synbiotics

Mechanistic basis and candidate strains: Probiotics are live bacteria which, when administered in adequate quantity, confer a health benefit on the host. The most investigated probiotic genera in the AD context are Lactobacillus, Bifidobacterium, Akkermansia, and Clostridiales species. Their putative mechanisms of action in AD include competitive exclusion of pathobionts; restoration of intestinal barrier integrity through upregulation of tight junction proteins (occludin, claudin-3, and ZO-1); attenuation of systemic and neuroinflammation through modulation of Toll-like receptor signaling; enhancement of SCFA production; and augmentation of neurotrophic factor expression [122,123].

Lactobacillus rhamnosus JB-1 has been shown to diminish anxiety-like behavior and normalize hippocampal GABA receptor expression in healthy mice, an effect mediated through the vagus nerve [99]. In APP/PS1 transgenic mice, concomitant memantine and Lactobacillus plantarum treatment attenuated cognitive impairment [124], and the multistrain probiotic formulation SLAB51 reduced NLRP3 inflammasome activation in a triple-transgenic model [123]. One mucin-degrading symbiont, Akkermansia muciniphila, is dramatically reduced in abundance in AD patients and has emerged as a particularly promising treatment candidate. Pasteurized (heat-killed) A. muciniphila preparations retain the bioactive Amuc_1100 outer membrane protein, which triggers TLR2 signaling to improve intestinal barrier function, reduce metabolic endotoxemia, and inhibit neuroinflammatory gene programs in microglia [125].

Several small randomized controlled trials have examined probiotic supplementation in patients with MCI or AD, with heterogeneous strains, doses, and durations. A systematic review of randomized trials of probiotic supplementation in AD highlighted considerable heterogeneity in strains, doses, durations, and outcome measures, which precludes firm conclusions [126]. Evidence is not limited to clinical AD and MCI populations: a randomized, double-blind, placebo-controlled, multicenter trial of Bifidobacterium bifidum BGN4 and Bifidobacterium longum BORI in sixty-three community-dwelling older adults without dementia found significant improvements in cognitive test performance and mood alongside compositional shifts in gut microbiota after twelve weeks of supplementation, suggesting that probiotic intervention may offer cognitive benefit earlier in the ageing trajectory, before clinical impairment is established [127].

Synbiotics, formulations combining probiotics with their corresponding prebiotic substrates, have shown additive or synergistic effects compared with either component alone in animal models, although human-trial data in AD populations remain scarce. In stressed adults without dementia, Lactobacillus plantarum P8 improved stress, anxiety, memory, and cognition in a randomized, placebo-controlled trial [128], but evidence for synbiotic interventions in MCI and AD remains limited.

### 9.3. Fecal Microbiota Transplantation

Rationale and preclinical data: FMT involves transfer of processed fecal material from a carefully selected healthy donor to a recipient in order to establish a diversified and functionally competent gut microbiome. The conceptual basis for FMT in AD rests on the germ-free and antibiotic-depletion studies described earlier, which indicate that the microbiota can influence key pathological hallmarks of AD in rodent models [17,18]. As described in Section 4.2, Harach and colleagues showed that germ-free APPPS1 transgenic mice exhibited significantly decreased cerebral amyloid deposits compared with their conventionally colonized counterparts, and that colonization with microbiota from AD-transgenic donors restored amyloid pathology in germ-free recipients, whereas colonization with wild-type microbiota produced only a partial increase; these results indicate that the microbiota can influence amyloid pathology in this familial AD transgenic model [17].

Later studies demonstrated that FMT from young, healthy donors to aged, cognitively impaired mice reversed age-related microbiome dysbiosis, reduced hippocampal neuroinflammation, and improved spatial and working memory performance to levels comparable with young control animals. These benefits were accompanied by increased fecal SCFA concentrations and restoration of occludin and ZO-1 tight junction protein expression [105]. In the 3xTg-AD model, FMT from healthy donors markedly reduced Aβ and tau pathology, decreased microglial reactivity, and increased hippocampal BDNF and synaptophysin expression. Shotgun metagenomic sequencing of recipient feces verified engraftment of donor taxa, especially SCFA producers, and metabolomic profiling revealed normalization of tryptophan and indole metabolite profiles [19].

Clinical feasibility and early-phase evidence: FMT for neurological indications remains at an earlier stage of development than for gastrointestinal indications such as recurrent Clostridioides difficile infection, where a landmark randomized trial established that FMT achieves cure rates exceeding 90%, far surpassing standard antibiotic therapy [129]. Safety profiles are generally favorable in major case series and RCTs for gastrointestinal indications, although serious adverse events, mostly related to aspiration or transfer of donor-derived pathogens, have been reported, and long-term safety data in older adults with dementia are lacking. Evidence for FMT in AD itself is limited to case reports and small uncontrolled or non-randomized studies: a single case report described rapid symptomatic improvement [130], and a small comparison in patients with cognitive decline and Clostridioides difficile infection reported cognitive improvement after FMT [84]. These observations cannot establish efficacy.

Registered FMT trials in AD are few, early-phase, and small. A phase 1 feasibility study of oral FMT (NCT03998423) was terminated early, an FMT capsule study in approximately 30 patients with clinically diagnosed AD is under way in Shanghai (FMT-AD, NCT06920212), and a single-arm phase 1 pilot of colonoscopic FMT (NCT07332260) plans to enroll about 10 participants [131,132]; none has yet reported efficacy outcomes. Critical issues for the clinical use of FMT in AD include donor selection criteria (optimal age, cognitive profile, and microbiome composition), standardization of preparation and delivery route (oral capsule versus colonoscopic versus nasoduodenal administration), duration and frequency of administration, and regulatory classification across different jurisdictions.

### 9.4. Drug and Microbiota-Targeted Approaches

Antibiotics, bacteriophages, and precision microbiome control: Broad-spectrum antibiotic treatment diminishes gut microbial diversity; in APPswe/PS1dE9 mice, it reduced Aβ plaque deposition and altered microglial morphology and cytokine profiles [18], which is why antibiotics are used experimentally to test microbiota dependence. Because antibiotics are non-selective and carry risks such as resistance and Clostridioides difficile overgrowth, they are research tools rather than candidate therapies; more selective approaches, including bacteriophages and precision microbiome editing, remain experimental and untested in AD.

Supplementation with SCFAs and butyrate prodrugs: Given the importance of butyrate deficiency in the pathogenic cascade from gut dysbiosis to neuroinflammation in AD, direct supplementation with butyrate or use of butyrate prodrugs has been investigated as a treatment option. In APPPS1-21 mice at an advanced stage of disease, the histone deacetylase inhibitor sodium butyrate improved associative memory [133]. Butyrate prodrugs with improved oral bioavailability are of interest, but clinical evidence in AD is lacking.

Regulation of the tryptophan pathway: The tryptophan–kynurenine–serotonin axis represents a key intersection of microbial metabolism, immunological control, and neurochemistry in AD. In AD, microbial dysbiosis correlates with increased indoleamine 2,3-dioxygenase 1 (IDO1) activity in the gut and brain, shunting tryptophan metabolism toward the neurotoxic kynurenine pathway and away from serotonin and indole synthesis; this leads to higher concentrations of quinolinic acid, an endogenous NMDA receptor agonist and excitotoxin, in hippocampal tissue [134]. Similarly, indole supplementation via dietary precursors (tryptophan and indole-3-carbinol) or direct indole molecules has shown anti-inflammatory effects in microglial cell lines and decreased Aβ aggregation in Caenorhabditis elegans AD models. Because more than 90% of ingested tryptophan is routed through the kynurenine pathway and only a small fraction is converted by gut bacteria to indole derivatives, ordinary dietary sources of tryptophan (oats, bananas, dried prunes, milk, tuna, poultry, and legumes) and their consistency of intake may be a more directly actionable nutritional lever than pharmacological IDO1 inhibition for shifting this balance in an ageing population [135].

Pharmacological approaches as complementary strategies: While nutritional and microbiome-targeted approaches directly modify the gut ecosystem, pharmacological strategies can modulate downstream gut-regulated pathways (e.g., incretin signaling and kynurenine metabolism) and may serve as complementary tools once their risk–benefit profiles are established.

## 10. Clinical Evidence and Clinical Trials

Translation of preclinical gut–brain-axis discoveries into therapeutic strategies for AD is at a pivotal juncture. This section focuses on ongoing and recently completed clinical trials that directly target the gut microbiome or gut-regulated pathways implicated in AD. Consistent with the nutritional focus of this review, we first summarize clinical trials of dietary patterns, prebiotics, probiotics, synbiotics, and fecal microbiota transplantation. Pharmacological trials that engage gut-regulated pathways (e.g., GLP-1 receptor agonists) are then discussed as complementary approaches, with emphasis on their mechanistic links to the gut–brain axis rather than as standalone AD therapies (Table 1).

### 10.1. Ongoing and Recently Completed Trials

#### 10.1.1. Prebiotic Interventions

The PRECODE trial (NCT06433037) is a four-arm, randomized, double-blind, placebo-controlled study of 26 weeks of supplementation with chicory inulin (12 g/day), resistant dextrin, or a seaweed polysaccharide versus maltodextrin placebo in 164 adults aged 60–79 years with subjective cognitive decline. The primary outcome is working-memory-related brain activation on functional MRI together with two-back task performance [136]. Because participants are at risk rather than biomarker-confirmed AD, the trial addresses prevention along the AD continuum rather than treatment of AD. Results are not yet available.

#### 10.1.2. Pharmacological Trials Targeting Gut-Regulated Pathways

The phase III trials EVOKE and EVOKE+ (NCT04777396 and NCT04777409) randomized approximately 3800 participants with biomarker-confirmed early symptomatic AD to oral semaglutide 14 mg/day or placebo for up to 156 weeks; the primary endpoint was change in the Clinical Dementia Rating–Sum of Boxes (CDR-SB) from baseline to week 104 [137,138]. Semaglutide did not slow clinical progression: in EVOKE, the estimated difference in CDR-SB change was −0.08 (95% CI −0.35 to 0.20; *p* = 0.57), and mean CDR-SB changes were 2.3 (EVOKE) and 2.2 (EVOKE+) with semaglutide versus 2.3 and 2.1 with placebo [138]. These negative results do not support GLP-1 receptor agonism as a disease-modifying strategy in AD, despite the mechanistic rationale linking GLP-1 signaling, gut microbiome modulation, and neuroinflammation.

We identified no registered, completed trial of a kynurenine pathway-targeted agent in AD; this pathway therefore remains supported by preclinical and observational evidence only.

Together, these registered trials show that gut-targeted intervention research in AD is at an early stage: the only large biomarker-anchored trials reported to date (EVOKE and EVOKE+) were negative, whereas microbiome-directed nutritional and FMT trials remain small, early-phase, or conducted in at-risk populations.

These completed and ongoing trials make clear that the field has moved well beyond mechanistic plausibility into active clinical testing, but they equally make clear that the evidence base remains fragmented across small, short, and methodologically diverse studies. The next section examines the broader limitations that constrain interpretation of this literature.

## 11. Limitations and Challenges in Gut–Brain-Axis Research

The clinical trial landscape reviewed above demonstrates real momentum, but interpreting it, and the wider mechanistic literature, requires confronting a series of structural limitations that recur across nearly every study design discussed in this review.

### 11.1. Inter-Individual Variability

The structure and function of microbial communities are significantly influenced by diet. While daily microbial responses to nutrition are highly individualized, microbiome composition depends on many days of dietary history and is more closely linked to overall food choices than to nutrient profiles in isolation [139]. Different individuals may respond differently to the same diet, and personalized nutrition has been increasingly recommended over “one size fits all” approaches. Environmental living conditions also substantially shape intestinal microbiota composition: living conditions, antibiotic and medication use, geographic location, lifestyle choices, household size, and even pet ownership all contribute. Alcohol and tobacco use have similarly been shown to alter gut microbiome diversity and composition [140]. This extreme variation makes establishing universal “healthy” or “AD-associated” microbial signatures fundamentally difficult and complicates cross-study comparison.

It is also difficult to disentangle whether the microbiota contributes to AD pathogenesis or whether AD itself causes microbial dysbiosis, since age-related microbiome remodeling and AD-associated microbial alterations overlap substantially [141]. The apparent links between microbial dysbiosis and AD may be exaggerated by the fact that many gut–brain-axis studies do not adequately control for biological age as distinct from chronological age [142].

Genetic variation represents a further significant source of inter-individual heterogeneity. The apolipoprotein E (APOE) genotype, the most significant genetic risk factor for late-onset AD, affects gut microbial composition independently of AD status; overall, the data point to a connection between gut microbiota composition and APOE genotype, with loss of SCFA- and butyrate-producing bacteria observed in APOE4 carriers, which may help explain how the APOE4 allele influences neuropathology [143]. Arguably, the most operationally difficult source of inter-individual heterogeneity is dietary pattern itself. Short-term microbiome variation is mostly driven by diet, yet the majority of studies either use inaccurate dietary recall tools, fail to standardize dietary intake before sampling, or omit dietary assessment entirely. The extent to which nutrition influences gut–brain outcomes as a confounder, mediator, or independent predictor in AD research is therefore rarely thoroughly investigated [144].

### 11.2. Methodological Inconsistencies

The gut–brain-axis field faces methodological challenges, arising from the use of animal models, sequencing methods, microbiome sampling protocols, and confounding environmental factors, that restrict the reproducibility and comparability of findings across laboratories and study populations [145]. Extensive use of DNA sequencing to characterize gut microbiota composition has revealed numerous associations between microbiota and host characteristics, including brain structure, function, and behavior. However, these correlations frequently lack follow-up validation and are inconsistent across observational studies, a discrepancy that hinders biological interpretation and clinical translation by making it more difficult to construct mechanistic investigations demonstrating causal links, and by causing validation studies to lag behind initial associative findings [146].

Most of the research is conducted using fecal samples, which are unlikely to fully reflect microbial activity within the gut itself, particularly in the small intestine or at the mucosal surface [147]. Protocols for collecting fecal samples vary considerably: the use of stabilizing buffers, storage conditions, time to storage, and fresh versus frozen handling all influence measured microbiome composition and introduce pre-analytical bias. Most clinical trials lack standardized bowel preparation and fail to account for concurrent probiotic, prebiotic, antibiotic, or laxative use, each of which substantially affects gut microbial composition [148].

The most widely used technique for examining the gut microbiome in AD research remains 16S ribosomal RNA (rRNA) amplicon sequencing. However, significant variation between studies in primer selection, targeted hypervariable regions (V1–V9), sequencing depth, and reference databases produces conflicting taxonomic results and complicates meta-analysis. Shotgun metagenomics, which delivers substantially more biological detail and can identify bacteria at the strain level while providing functional insight, remains less common because of its greater cost and analytical complexity [149].

Many studies also do not adequately address possible confounding influences, such as housing conditions, diet, and cage effects in animal models, that complicate interpretation and limit generalizability, and the validity of certain behavioral assessments and tests is often assumed rather than demonstrated [150]. Confounders are frequently selected based on prior research rather than systematic evaluation: age, sex, BMI, diet, antibiotic use, and gastrointestinal symptoms are common confounders in microbiome gut–brain-axis studies, while alcohol use, despite being a recognized microbiota-related confounder, has received insufficient attention. Other factors particularly relevant to AD include APOE genotype, medication use (including proton-pump inhibitors, metformin, and anticholinergics), comorbidities such as diabetes, institutionalization and dietary change, and bowel habit; however, the paucity of research on microbiota–host relationships in specific populations means that there are few established references to guide confounder selection [146].

### 11.3. Translational Limitations

The suitability of animal models for human disease, although rarely debated explicitly, is crucial to interpreting these investigations. Beyond differences in gut structure, neuroanatomy, microbiota composition, and behavior between humans and mice, there are also issues in translating claims about stress, anxiety, and depression from animal models into clinical practice: behaviors such as fearfulness and timidity, which may be typical for mice, are not necessarily comparable to human presentations, and vice versa; thus, rodents cannot provide the self-report data that underpin much human psychiatric assessment [150].

At the phylum, genus, and species level, the microbiome of mice used in AD gut–brain research differs substantially from that of humans. Both are dominated by Bacteroidetes and Firmicutes, but specific genus- and species-level compositions differ considerably, and many clinically significant human commensals lack strict mouse equivalents. Mice also develop microbiomes that are not comparable to those of free-living humans exposed to complex ambient microbial communities, since they are coprophagic, lack a true appendix, have different intestinal transit times, and are typically housed in controlled, largely sterile environments [151].

Transgenic mice that overexpress human genes associated with familial AD and develop amyloid plaques are the most widely used experimental models, but since neurofibrillary tangles and amyloid plaques are also present together in human AD, these models only partially represent the disease. This limitation reduces the success of AD clinical trials, since results from animal models are often extrapolated to humans prematurely. Models such as APP/PS1, 3xTg-AD, and 5xFAD, based on familial AD mutations that account for less than 5% of human cases, replicate amyloid and tau pathology in an accelerated, non-physiological manner. The majority of human AD, by contrast, is late-onset and sporadic, involving intricate gene–environment–microbiome interactions that current models cannot fully reproduce, thus limiting the generalizability of gut–brain-axis findings derived from them [152].

The lack of standardized, validated biomarkers for gut-mediated neuroinflammation and neurodegeneration that could serve as surrogate endpoints in clinical studies further impedes clinical translation. Short follow-up periods (typically weeks to months), small sample sizes insufficiently powered to detect cognitive outcomes, heterogeneous patient selection criteria, and the lack of concurrent neuroimaging or CSF biomarker assessment remain the principal limitations of current probiotic, prebiotic, and FMT trials in AD and MCI populations. Definitive proof of treatment success therefore remains elusive in the absence of established molecular biomarkers linking gut intervention to downstream alteration of AD pathology [28].

Taken together, these limitations do not undermine the mechanistic case assembled in earlier sections, but they do explain why that case has not yet translated into definitive clinical evidence. The final section considers how the field might be structured going forward to close this gap.

## 12. Future Perspectives and Research Directions

The limitations outlined above point toward specific, addressable directions for the field. This section considers four such directions: precision medicine approaches, multi-omics integration, longitudinal and interventional study design, and the broader translational agenda.

### 12.1. Precision Medicine and Personalized Microbiome Approaches

Beyond its known connections to altered microbial community structure in disease, the gut microbiome is becoming increasingly recognized as a biomarker for disease phenotype, prognosis, and treatment response. For individualized diagnosis, including disease type, severity, and prognosis, microbial fingerprints offer accurate, non-invasive, and affordable assessment methods. The microbiome also plays a crucial role in drug availability, efficacy, and toxicity through its effects on the metabolism of numerous compounds, making it relevant to tailored treatment design; its adaptability supports targeted, microbiome-based therapies that may improve outcomes across multiple conditions by customizing therapy to an individual’s microbial composition [153].

Machine-learning algorithms trained on combined microbiome, genomic, metabolomic, and clinical data can now predict individual responses to dietary and probiotic therapies with substantially greater accuracy than population averages. A seminal study demonstrated that postprandial glycemic responses, a proxy for metabolic microbiome function, are highly individualized and can be predicted using microbiome-informed personalized models, a framework that could, in principle, be extended to neurological outcomes [154]. Similar approaches should be applied in future research to determine which AD patients, based on host genetic background and baseline microbiome configuration, will benefit from particular probiotic formulations, dietary changes such as the MIND diet, or prebiotic supplementation [155].

A key component of precision microbiome treatment for AD is live biotherapeutic products, regulatory-grade microbial formulations that differ from traditional probiotics. Unlike conventional probiotics, live biotherapeutic products can be engineered to provide specific neuroactive metabolites or anti-inflammatory compounds, are subject to pharmaceutical-grade quality control, and permit strain-specific dosing. Ongoing early-phase studies of microbial consortia designed to increase SCFA production, decrease LPS bioavailability, or improve conversion of tryptophan to serotonin represent an important step toward clinical translation [105].

Psychobiotics are a specialized group of probiotics that influence brain function and behavior, principally through interaction with the gut–brain axis. For a probiotic to be considered a psychobiotic, it must produce neuroactive substances, particularly neurotransmitters such as GABA and serotonin, which play key roles in regulating mood and cognitive function; these substances may also help lower inflammation and oxidative stress, both implicated in neurodegenerative disorders. There is growing interest in using probiotics and psychobiotics as potential treatments for neurological and psychiatric conditions, including AD. However, before they can be widely adopted clinically, researchers need a clearer understanding of which specific strains are most effective, how individual differences affect outcomes, and how treatment should be tailored to different disease stages. The field is likely to move toward more personalized approaches that combine psychobiotics with other therapies within broader, multi-faceted treatment strategies [156].

### 12.2. Multi-Omics Integration

Over the past decade, omics technologies have advanced considerably in helping researchers interpret the complex makeup of the metabolome, lipidome, and proteome. As advanced tools for studying biological traits, they have made it substantially easier to uncover the underlying molecular changes that drive complex diseases such as AD [157]. Multi-omics integration, combining multiple omics layers, gives researchers a more comprehensive picture of disease pathophysiology and a better understanding of the relationships between biomarkers. Single-layer analyses capture only a portion of disease biology, whereas multi-omics integration prioritizes pathways, resolves heterogeneity, and connects genomic variation to downstream RNA and protein effects. These frameworks support more reliable blood and CSF biomarker panels, higher-confidence treatment targets, and subtype delineation within AD [158].

Metabolomics makes it possible to monitor variation in a pool of metabolites that reflect changes downstream of genomic, transcriptomic, and proteomic fluctuations, providing a precise biochemical profile of an organism in both health and disease and helping to clarify changes in the intricate molecular networks implicated in AD [159]. Plasma and CSF metabolomics have identified several gut microbiome-derived metabolites, including TMAO, secondary bile acids, tryptophan catabolites, and uremic toxins, as disease-associated signals in AD. Future multi-omics studies should combine concurrent fecal, plasma, and CSF metabolomics with microbiome sequencing and neuroimaging biomarkers (amyloid PET, tau PET, and FDG-PET) in order to build quantitative models of metabolite-mediated gut–brain-axis dysfunction spanning from the intestine to the synapse [160].

### 12.3. Longitudinal and Interventional Studies

Because most of the existing research is cross-sectional and observational, relatively little is firmly established about the temporal and mechanistic role of the gut–brain axis in AD. Most current research demonstrates associations between gut microbiota composition and AD pathophysiology without establishing temporal or causal relationships. Longitudinal human studies are therefore clearly needed to better understand how microbiome changes relate to disease onset and progression. Future research should prioritize longitudinal cohort designs with repeated assessments over time to evaluate changes in gut microbiota alongside established AD biomarkers; combining microbiome analysis with clinical and biological measurements, such as biomarkers of tau and amyloid pathology, may clarify the pathways connecting gut dysbiosis to dementia, enhance understanding of disease causation, and facilitate development of microbiome-targeted treatment approaches [161].

Interventional studies in AD are increasingly using fluid-based biomarkers to improve trial design, building on this need for longitudinal evidence. Because cognitive outcomes typically require larger samples and longer follow-up to detect meaningful improvement, relying on cognitive measures alone remains limiting. As a result, validated biomarkers such as the amyloid-β 42/40 ratio, phosphorylated tau, and neurofilament light are increasingly used as primary or co-primary endpoints alongside cognitive evaluation. Incorporating these biomarkers into trial frameworks may allow biological responses to be detected with greater sensitivity and improve participant characterization and monitoring, potentially improving assessment of treatment effectiveness and supporting development of more targeted AD treatments [162].

Dietary intervention remains a safe and readily applicable method for modulating the gut–brain axis in AD. In observational studies, the MIND diet, a Mediterranean–DASH hybrid rich in omega-3 fatty acids, polyphenols, and prebiotic fibers, has shown promise for lowering AD risk and delaying cognitive decline. Future interventional trials should incorporate gut microbiome profiling as a primary mechanistic outcome, both to determine which dietary patterns are best suited to particular baseline microbiome configurations and to directly evaluate the extent to which diet-induced microbiome changes mediate cognitive benefit.

Although current clinical translation remains focused mostly on nutritional approaches, there is considerable interest in developing microbiome-targeted pharmacological techniques, building on this expanding evidence base. Important priorities for advancing the field include expanding the comparatively small number of randomized controlled trials assessing dietary, prebiotic, and probiotic interventions in AD, and improving the translational relevance of preclinical models, including germ-free or genetically homogeneous mice, so that they better reflect heterogeneous human populations. Addressing these issues will strengthen the evidence base supporting development of more effective microbiome-informed treatment approaches for AD and other neurodegenerative illnesses.

## 13. Conclusions

The evidence reviewed here supports a biologically plausible association between the microbiota–gut–brain axis and AD-related amyloid, tau, and neuroinflammatory processes. Preclinical studies indicate that experimental manipulation of the microbiota can modify these phenotypes in selected animal models, whereas human studies remain heterogeneous and primarily associational. In rodent models, loss of intestinal barrier integrity and the translocation of lipopolysaccharide, bacterial amyloids, and other dysbiosis-associated metabolites are proposed to activate innate immune signaling pathways, notably TLR4–NF-κB and NLRP3–pyroptosis, which may predispose microglia to a chronic pro-inflammatory phenotype and impair their ability to clear Aβ. Similarly, dysbiosis-associated depletion of short-chain fatty acids and diversion of tryptophan metabolism towards kynurenine pathway products may converge on tau hyperphosphorylation, synaptic dysfunction, and reduced neurotrophic signaling; direct evidence for this sequence in human AD is currently lacking.

Preclinical germ-free and fecal-transfer models provide direct evidence within transgenic and antibiotic-depletion rodent systems, while observational human data and an increasing amount of Mendelian randomization evidence are converging on, but do not by themselves establish, a comparable contribution in human disease. Taken together, these independent lines of evidence support an active, mechanistically contributory role for the gut microbiome in AD pathophysiology in preclinical models, though no single line of human evidence is sufficient alone to establish directionality.

Early clinical studies have reported preliminary cognitive, biomarker, or microbiome-related signals, but these findings remain insufficient to establish clinical efficacy. Modest benefits have been reported in small, heterogeneous trials of probiotic and synbiotic supplementation and in observational studies of Mediterranean and MIND dietary patterns, whereas no registered fecal microbiota transplantation trial in AD has reported efficacy outcomes. Pharmacological strategies targeting the kynurenine pathway remain untested in registered AD trials, and the phase 3 EVOKE and EVOKE+ trials of oral semaglutide, a GLP-1 receptor agonist, did not slow clinical progression. At the same time, the field still faces the limitations outlined in Section 11: large interindividual variability in microbiome composition, inconsistencies in sequencing and reporting methods; imperfect translatability of transgenic and germ-free animal models to sporadic late-onset human disease; and a clinical trial landscape still dominated by small, short-duration studies with heterogeneous outcome measures.

Addressing this disconnect between mechanistic plausibility and clinical utility will require longitudinal human cohorts tracking microbiome composition, in addition to fluid and imaging biomarkers of amyloid and tau pathology; adequately powered interventional trials with standardized, biomarker-anchored endpoints; and precision medicine frameworks integrating host genotype (including APOE status), microbiome enterotype, and dietary exposure. If these prerequisites are met, the microbiota–gut–brain axis could be evaluated more rigorously as a potential target for AD prevention or treatment.

## Figures and Tables

**Table 1:** Registered gut–brain-axis-targeted trials in Alzheimer’s disease.

Intervention Class	Trial/Study (Registry)	Primary Endpoint	Biomarker-Confirmed AD vs. Clinical Diagnosis	Design and Population	Duration	Key Outcomes (Prespecified)	Effect Size/Statistical Significance	Clinical Relevance	Main Limitations	Status
Prebiotic (inulin, resistant dextrin, seaweed polysaccharide)	PRECODE (NCT06433037) [136]	Working-memory-related fMRI activation and 2-back performance	Subjective cognitive decline (at risk); not biomarker-confirmed AD	RCT, four-arm, double-blind, placebo-controlled; *n* = 164, aged 60–79; fiber vs. maltodextrin	26 weeks	Primary: fMRI working-memory activation and 2-back performance. Secondary: Gut, immune, glycemic and mood measures. Results not yet published.	Not yet reported	Tests prebiotic fiber for prevention in at-risk older adults along the AD continuum.	Not an AD population; imaging-based primary outcome; results pending.	Results pending
Fecal microbiota transplantation (FMT capsules)	FMT-AD (NCT06920212) [131]	Clinical safety and efficacy (registry detail limited)	Clinical diagnosis of AD (MMSE 3–26; MRI atrophy); not biomarker-confirmed	Interventional; *n* ≈ 30, aged 50–85; FMT capsules; single center (Shanghai)	Not specified in registry summary	Clinical safety, cognitive efficacy and gut microbiota change before vs. after FMT. Results not published.	Not yet reported	One of few registered FMT studies in patients with AD.	Small, single-center, clinical diagnosis; limited design detail; no efficacy data.	Active, not recruiting
GLP-1 receptor agonist (oral semaglutide)	EVOKE and EVOKE+ (NCT04777396; NCT04777409) [137,138]	Change in CDR-SB from baseline to week 104	Early symptomatic AD; biomarker confirmation required (amyloid PET or CSF)	Two phase III, randomized, double-blind, placebo-controlled trials; total *n* ≈ 3800 with early symptomatic AD; oral semaglutide 14 mg/day vs. placebo	156 weeks (primary analysis at week 104)	Primary: Change in CDR-SB at week 104. No slowing of cognitive or functional decline with semaglutide.	EVOKE: Difference −0.08 (95% CI −0.35 to 0.20; *p* = 0.57). Mean CDR-SB change 2.3 (EVOKE) and 2.2 (EVOKE+) with semaglutide vs. 2.3 and 2.1 with placebo.	Large, rigorous trials; negative result argues against GLP-1 receptor agonism as disease-modifying in early AD.	Gut–brain link is indirect; effects of longer treatment unknown.	Completed; results published
Fecal microbiota transplantation (oral)	Oral FMT feasibility study (NCT03998423) [132]	Safety and feasibility; engraftment by 16S rRNA sequencing	Alzheimer’s clinical syndrome (registry detail limited)	Phase 1, oral FMT feasibility study; single site (University of Wisconsin); reported enrolment ≈ 5	1-year cognitive follow-up (MoCA, RBANS)	Safety, feasibility, engraftment; exploratory MoCA and RBANS change. No results published.	Not reported	Early feasibility data for non-invasive FMT delivery in AD.	Terminated; very small sample; no efficacy conclusions possible.	Terminated

## Data Availability

No new data were created or analyzed in this study. Data sharing is not applicable to this article.

## Abbreviations

The following abbreviations are used in this manuscript:

5-HT	5-hydroxytryptamine (serotonin)
Aβ	Amyloid-beta
AD	Alzheimer’s disease
AHAD	American Heart Association diet
AhR	Aryl hydrocarbon receptor
APOE	Apolipoprotein E
APP	Amyloid precursor protein
APPPS1	APPSWE/PS1 transgenic mouse model
ARIA	Amyloid-related imaging abnormalities
ARIA-E	ARIA with vasogenic oedema
ARIA-H	ARIA with microhemorrhage or superficial siderosis
BACE1	Beta-site APP cleaving enzyme 1
BBB	Blood–brain barrier
BDNF	Brain-derived neurotrophic factor
C99	99-amino-acid *C*-terminal fragment of APP
CA	Cornu ammonis (hippocampal subfield)
CDI	*Clostridioides difficile* infection
CDR-SB	Clinical Dementia Rating Sum of Boxes
CI	Confidence interval
CNS	Central nervous system
CSF	Cerebrospinal fluid
DA	D-lactic acid
DAO	Diamine oxidase
DASH	Dietary Approaches to Stop Hypertension
EGCG	Epigallocatechin-3-gallate
ENS	Enteric nervous system
FMT	Fecal microbiota transplantation
FOS	Fructo-oligosaccharide
GABA	Gamma-aminobutyric acid
GLP-1	Glucagon-like peptide-1
GSK-3	Glycogen synthase kinase-3
HPA	Hypothalamic–pituitary–adrenal
IDO	Indoleamine 2,3-dioxygenase
IFN-γ	Interferon-gamma
IL	Interleukin
IQR	Interquartile range
KD	Ketogenic diet
KP	Kynurenine pathway
KYNA	Kynurenic acid
LBP	LPS-binding protein
LPS	Lipopolysaccharide
LTP	Long-term potentiation
MAM	Mitochondria-associated membrane
MCI	Mild cognitive impairment
MIND	Mediterranean–DASH Intervention for Neurodegenerative Delay
MMSE	Mini-Mental State Examination
MoCA	Montreal Cognitive Assessment
MRI	Magnetic resonance imaging
NF-κB	Nuclear factor kappa B
NLRP3	NLR family pyrin domain-containing 3
NMDA	N-methyl-D-aspartate
OMV	Outer membrane vesicle
PET	Positron emission tomography
QUIN	Quinolinic acid
RBANS	Repeatable Battery for the Assessment of Neuropsychological Status
RCT	Randomised controlled trial
SCFA	Short-chain fatty acid
TDO	Tryptophan 2,3-dioxygenase
TLR	Toll-like receptor
TMAO	Trimethylamine N-oxide
TNF-α	Tumour necrosis factor-alpha
TPH1	Tryptophan hydroxylase 1
ZO-1	Zonula occludens-1

## References

[1] Nichols E., Steinmetz J.D., Vollset S.E., Fukutaki K., Chalek J., Abd-Allah F., Abdoli A., Abualhasan A., Abu-Gharbieh E., Akram T.T. (2022). Estimation of the global prevalence of dementia in 2019 and forecasted prevalence in 2050: An analysis for the Global Burden of Disease Study 2019. Lancet Public Health.

[2] Livingston G., Huntley J., Liu K.Y., Costafreda S.G., Selbæk G., Alladi S., Ames D., Banerjee S., Burns A., Brayne C. (2024). Dementia prevention, intervention, and care: 2024 report of the Lancet Standing Commission. Lancet.

[3] Vishnumukkala T., Gopalakrishna P.K., Jagadeesan S., Chiroma S.M., Mohd Nor N.H., Baharuldin M.T.H., Thomas W., Mohd Moklas M.A. (2024). Herbal Medicine: A Promising Approach for the Treatment of Alzheimer’s Disease. Int. J. Ayurveda Pharma Res..

[4] van Dyck C.H., Swanson C.J., Aisen P., Bateman R.J., Chen C., Gee M., Kanekiyo M., Li D., Reyderman L., Cohen S. (2023). Lecanemab in early Alzheimer’s disease. N. Engl. J. Med..

[5] Sims J.R., Zimmer J.A., Evans C.D., Lu M., Ardayfio P., Sparks J., Wessels A.M., Shcherbinin S., Wang H., Monkul Nery E.S. (2023). Donanemab in early symptomatic Alzheimer disease: The TRAILBLAZER-ALZ 2 randomized clinical trial. JAMA.

[6] Haeberlein S.B., Aisen P., Barkhof F., Chalkias S., Chen T., Cohen S., Dent G., Hansson O., Harrison K., von Hehn C. (2022). Two randomized phase 3 studies of aducanumab in early Alzheimer’s disease. J. Prev. Alzheimer’s Dis..

[7] Cryan J.F., O’RIordan K.J., Cowan C.S.M., Sandhu K.V., Bastiaanssen T.F.S., Boehme M., Codagnone M.G., Cussotto S., Fulling C., Golubeva A.V. (2019). The microbiota-gut-brain axis. Physiol. Rev..

[8] Erny D., de Angelis A.L.H., Jaitin D., Wieghofer P., Staszewski O., David E., Keren-Shaul H., Mahlakoiv T., Jakobshagen K., Buch T. (2015). Host microbiota constantly control maturation and function of microglia in the CNS. Nat. Neurosci..

[9] Cattaneo A., Cattane N., Galluzzi S., Provasi S., Lopizzo N., Festari C., Ferrari C., Guerra U.P., Paghera B., Muscio C. (2017). Association of brain amyloidosis with pro-inflammatory gut bacterial taxa and peripheral inflammation markers in cognitively impaired elderly. Neurobiol. Aging.

[10] Sender R., Fuchs S., Milo R. (2016). Revised estimates for the number of human and bacteria cells in the body. PLoS Biol..

[11] Turner J.R. (2009). Intestinal mucosal barrier function in health and disease. Nat. Rev. Immunol..

[12] Chen S.G., Stribinskis V., Rane M.J., Demuth D.R., Gozal E., Roberts A.M., Jagadapillai R., Liu R., Choe K., Shivakumar B. (2016). Exposure to the functional bacterial amyloid protein curli enhances alpha-synuclein aggregation in aged Fischer 344 rats and Caenorhabditis elegans. Sci. Rep..

[13] Vogt N.M., Kerby R.L., Dill-McFarland K.A., Harding S.J., Merluzzi A.P., Johnson S.C., Carlsson C.M., Asthana S., Zetterberg H., Blennow K. (2017). Gut microbiome alterations in Alzheimer’s disease. Sci. Rep..

[14] Liu P., Wu L., Peng G., Han Y., Tang R., Ge J., Zhang L., Jia L., Yue S., Zhou K. (2019). Altered microbiomes distinguish Alzheimer’s disease from amnestic mild cognitive impairment and health in a Chinese cohort. Brain Behav. Immun..

[15] Ferreiro A.L., Choi J., Ryou J., Newcomer E.P., Thompson R., Bollinger R.M., Hall-Moore C., Ndao I.M., Sax L., Benzinger T.L.S. (2023). Gut microbiome composition may be an indicator of preclinical Alzheimer’s disease. Sci. Transl. Med..

[16] Jemimah S., Chabib C.M.M., Hadjileontiadis L., AlShehhi A. (2023). Gut microbiome dysbiosis in Alzheimer’s disease and mild cognitive impairment: A systematic review and meta-analysis. PLoS ONE.

[17] Harach T., Marungruang N., Duthilleul N., Cheatham V., Mc Coy K.D., Frisoni G., Neher J.J., Fåk F., Jucker M., Lasser T. (2017). Reduction of Abeta amyloid pathology in APPPS1 transgenic mice in the absence of gut microbiota. Sci. Rep..

[18] Minter M.R., Zhang C., Leone V., Ringus D.L., Zhang X., Oyler-Castrillo P., Musch M.W., Liao F., Ward J.F., Holtzman D.M. (2016). Antibiotic-induced perturbations in gut microbial diversity influences neuro-inflammation and amyloidosis in a murine model of Alzheimer’s disease. Sci. Rep..

[19] Kim M.-S., Kim Y., Choi H., Kim W., Park S., Lee D., Kim D.K., Kim H.J., Choi H., Hyun D.-W. (2020). Transfer of a healthy microbiota reduces amyloid and tau pathology in an Alzheimer’s disease animal model. Gut.

[20] Colombo A.V., Sadler R.K., Llovera G., Singh V., Roth S., Heindl S., Monasor L.S., Verhoeven A., Peters F., Parhizkar S. (2021). Microbiota-derived short chain fatty acids modulate microglia and promote Aβ plaque deposition. eLife.

[21] Kearns R. (2024). Gut-brain axis and neuroinflammation: The role of gut permeability and the kynurenine pathway in neurological disorders. Cell Mol. Neurobiol..

[22] Lin C., Zhao S., Zhu Y., Fan Z., Wang J., Zhang B., Chen Y. (2019). Microbiota-gut-brain axis and toll-like receptors in Alzheimer’s disease. Comput. Struct. Biotechnol. J..

[23] Schoultz I., Keita Å.V. (2020). The intestinal barrier and current techniques for the assessment of gut permeability. Cells.

[24] Suzuki T. (2020). Regulation of the intestinal barrier by nutrients: The role of tight junctions. Anim. Sci. J..

[25] Buschmann M.M., Shen L., Rajapakse H., Raleigh D.R., Wang Y., Wang Y., Lingaraju A., Zha J., Abbott E., McAuley E.M. (2013). Occludin OCEL-domain interactions are required for maintenance and regulation of the tight junction barrier to macromolecular flux. Mol. Biol. Cell.

[26] He J., Liu Y., Li J., Zhao Y., Jiang H., Luo S., He G. (2023). Intestinal changes in permeability, tight junction and mucin synthesis in a mouse model of Alzheimer’s disease. Int. J. Mol. Med..

[27] Pei Y., Lu Y., Li H., Jiang C., Wang L. (2023). Gut microbiota and intestinal barrier function in subjects with cognitive impairments: A cross-sectional study. Front. Aging Neurosci..

[28] Kowalski K., Mulak A. (2019). Brain-gut-microbiota axis in Alzheimer’s disease. J. Neurogastroenterol. Motil..

[29] Vaziri Y., Olia J.B.H., Avci C.B., Nourazarian A. (2025). Molecular mechanisms of gut microbiota dysbiosis and metabolites in Alzheimer’s disease pathogenesis: Implications for precision therapeutics. Mol. Brain.

[30] Al-Sadi R., Boivin M., Ma T. (2009). Mechanism of cytokine modulation of epithelial tight junction barrier. Front. Biosci..

[31] Moonwiriyakit A., Pathomthongtaweechai N., Steinhagen P.R., Chantawichitwong P., Satianrapapong W., Pongkorpsakol P. (2023). Tight junctions: From molecules to gastrointestinal diseases. Tissue Barriers.

[32] Shaheen N., Miao J., Xia B., Zhao Y., Zhao J. (2025). Multifaceted role of microbiota-derived indole-3-acetic acid in human diseases and its potential clinical application. FASEB J..

[33] Abolhasani F.S., Vaghefinanekaran N., Yarahmadi A., Akrami S., Mirmahdavi S., Yousefi M.H., Afkhami H., Shafiei M. (2025). Outer membrane vesicles in gram-negative bacteria and its correlation with pathogenesis. Front. Immunol..

[34] Park A.M., Tsunoda I. (2022). Helicobacter pylori infection in the stomach induces neuroinflammation: The potential roles of bacterial outer membrane vesicles in an animal model of Alzheimer’s disease. Inflamm. Regen..

[35] Barrett K.E. (2020). Claudin-2 pore causes leak that breaches the dam in intestinal inflammation. J. Clin. Invest..

[36] Saito T., Hayashida H., Furugen R. (2007). Comment on: Cani et al. (2007) Metabolic endotoxemia initiates obesity and insulin resistance: Diabetes 56:1761-1772. Diabetes.

[37] Guo S., Nighot M., Al-Sadi R., Alhmoud T., Nighot P., Ma T.Y. (2015). Lipopolysaccharide regulation of intestinal tight junction permeability is mediated by TLR4 signal transduction pathway activation of FAK and MyD88. J. Immunol..

[38] Yamazaki Y., Shinohara M., Shinohara M., Yamazaki A., Murray M.E., Liesinger A.M., Heckman M.G., Lesser E.R., Parisi J.E., Petersen R.C. (2019). Selective loss of cortical endothelial tight junction proteins during Alzheimer’s disease progression. Brain.

[39] Müller L., Di Benedetto S. (2025). Bridging the brain and gut: Neuroimmune mechanisms of neuroinflammation and therapeutic insights. Front. Cell. Neurosci..

[40] Lei W., Cheng Y., Liu X., Gao J., Zhu Z., Ding W., Xu X., Li Y., Ling Z., Jiang R. (2025). Gut microbiota-driven neuroinflammation in Alzheimer’s disease: From mechanisms to therapeutic opportunities. Front. Immunol..

[41] Nasr El-Din W.A., Abdel Fattah I.O. (2024). L-arginine mitigates choroid plexus changes in Alzheimer’s disease rat model via oxidative/inflammatory burden and behavioral modulation. Tissue Cell.

[42] Gopalakrishna P.K., Che Mohd Nassir C.M.N., Anandan S., Hussan F., Dandala K.C.R., Sura S., Jagadeesan S., Mohd Moklas M.A., Vishnumukkala T., Hein Z.M. (2026). The Immuno-Glial Connectome in Alzheimer’s Disease: Integrating Central and Peripheral Inflammatory Networks. Cell. Mol. Neurobiol..

[43] Ta T.T., Dikmen H.O., Schilling S., Chausse B., Lewen A., Hollnagel J.O., Kann O. (2019). Priming of microglia with IFN-γ slows neuronal gamma oscillations in situ. Proc. Natl. Acad. Sci. USA.

[44] Qin R., Li C., Yuan X., Chen Y. (2025). Microbiome-targeted Alzheimer’s interventions via gut-brain axis. Front. Microbiol..

[45] Jha D., Bakker E.N.T.P., Kumar R. (2024). Mechanistic and therapeutic role of NLRP3 inflammasome in the pathogenesis of Alzheimer’s disease. J. Neurochem..

[46] Li Z., Gong C. (2025). NLRP3 inflammasome in Alzheimer’s disease: Molecular mechanisms and emerging therapies. Front. Immunol..

[47] He X.-F., Xu J.-H., Li G., Li M.-Y., Li L.-L., Pei Z., Zhang L.-Y., Hu X.-Q. (2020). NLRP3-dependent microglial training impaired the clearance of amyloid-beta and aggravated the cognitive decline in Alzheimer’s disease. Cell Death Dis..

[48] Van Zeller M., Dias D., Sebastião A.M., Valente C.A. (2021). NLRP3 inflammasome: A starring role in amyloid-β- and tau-driven pathological events in Alzheimer’s disease. J. Alzheimer’s Dis..

[49] Kakkar A., Singh H., Singh B.K., Kumar A., Mishra A.K., Chopra H. (2025). Neuroinflammation and Alzheimer’s disease: Unravelling the molecular mechanisms. J. Alzheimer’s Dis..

[50] Heston M.B., Hanslik K.L., Zarbock K.R., Harding S.J., Davenport-Sis N.J., Kerby R.L., Chin N., Sun Y., Hoeft A., Deming Y. (2023). Gut inflammation associated with age and Alzheimer’s disease pathology: A human cohort study. Sci. Rep..

[51] Lanz M., Janeiro M.H., Milagro F.I., Puerta E., Ludwig I.A., Pineda-Lucena A., Ramírez M.J., Solas M. (2022). Trimethylamine N-oxide (TMAO) drives insulin resistance and cognitive deficiencies in a senescence accelerated mouse model. Mech. Ageing Dev..

[52] Wang P., Mi Y., Yu H., Teng X., Jin S., Xiao L., Xue H., Tian D., Guo Q., Wu Y. (2024). Trimethylamine-N-oxide aggravated the sympathetic excitation in D-galactose induced aging rats by down-regulating P2Y12 receptor in microglia. BioMed. Pharmacother..

[53] Elkins M., Jain N., Tükel Ç. (2024). The menace within: Bacterial amyloids as a trigger for autoimmune and neurodegenerative diseases. Curr. Opin. Microbiol..

[54] Zeng J., Liao Z., Yang H., Wang Q., Wu Z., Hua F., Zhou Z. (2024). T cell infiltration mediates neurodegeneration and cognitive decline in Alzheimer’s disease. Neurobiol. Dis..

[55] Yang Q., Qiu Y., Ni J., Li H., Qing H. (2025). Deciphering T cell dynamics in Alzheimer’s disease pathogenesis: Insights and implications. Curr. Neuropharmacol..

[56] Jorfi M., Park J., Hall C.K., Lin C.-C.J., Chen M., von Maydell D., Kruskop J.M., Kang B., Choi Y., Prokopenko D. (2023). Infiltrating CD8+ T cells exacerbate Alzheimer’s disease pathology in a 3D human neuroimmune axis model. Nat. Neurosci..

[57] Huang R., Lin B.-B., Lu Z., Hao Y., Li C., Lin Z., Zhang Y., Wei N., Chen J. (2026). Identification of plasma inflammatory biomarkers for Alzheimer’s disease reveals IFN-γ as a regulator of ACSL1-mediated microglia phenotype. Front. Immunol..

[58] Liang Y., Xie S., He Y., Xu M., Qiao X., Zhu Y., Wu W. (2022). Kynurenine pathway metabolites as biomarkers in Alzheimer’s disease. Dis. Markers.

[59] Li G., Li S., Zhou W. (2025). Kynurenine pathway: A possible new mechanism for exercise in the prevention and treatment of Alzheimer’s disease. Front. Aging Neurosci..

[60] Argolo D.S., de Oliveira L.M.G., Guillemin G.J., Barreto G.E., Butt A.M., Costa S.L., Costa M.F.D. (2025). Tryptophan metabolism through the kynurenine pathway in glial cells. Neuroglia.

[61] Cao Q., Shen M., Li R., Liu Y., Zeng Z., Zhou J., Niu D., Zhang Q., Wang R., Yao J. (2025). Elucidating the specific mechanisms of the gut-brain axis: The short-chain fatty acids-microglia pathway. J. Neuroinflammation.

[62] Silva Y.P., Bernardi A., Frozza R.L. (2020). The role of short-chain fatty acids from gut microbiota in gut-brain communication. Front. Endocrinol..

[63] Wang H., Yang F., Gao Z., Cheng Z., Liang X. (2025). The gut-brain axis in Alzheimer’s disease: How gut microbiota modulate microglial function. Front. Aging.

[64] Sun J., Zhang Y., Kong Y., Ye T., Yu Q., Satyanarayanan S.K., Su K.-P., Liu J. (2022). Microbiota-derived metabolite indoles induced aryl hydrocarbon receptor activation and inhibited neuroinflammation in APP/PS1 mice. Brain Behav. Immun..

[65] Nonogaki K., Kaji T. (2025). Tryptophan hydroxylase 1 regulates tryptophan and its metabolites. Int. J. Mol. Sci..

[66] Liu N., Sun S., Wang P., Sun Y., Hu Q., Wang X. (2021). The mechanism of secretion and metabolism of gut-derived 5-hydroxytryptamine. Int. J. Mol. Sci..

[67] Xia Y., Xiao Y., Wang Z.-H., Liu X., Alam A.M., Haran J.P., McCormick B.A., Shu X., Wang X., Ye K. (2023). Bacteroides fragilis in the gut microbiomes of Alzheimer’s disease activates microglia and triggers pathogenesis in neuronal C/EBPβ transgenic mice. Nat. Commun..

[68] Zhan X., Stamova B., Jin L.W., DeCarli C., Phinney B., Sharp F.R. (2016). Gram-negative bacterial molecules associate with Alzheimer disease pathology. Neurology.

[69] Zhao Y., Cong L., Lukiw W.J. (2017). Lipopolysaccharide (LPS) accumulates in neocortical neurons of Alzheimer’s disease brain and impairs transcription in human neuronal-glial primary co-cultures. Front. Aging Neurosci..

[70] Zhao Y., Jaber V., Lukiw W.J. (2017). Secretory products of the human GI tract microbiome and their potential impact on Alzheimer’s disease: Detection of lipopolysaccharide (LPS) in Alzheimer’s disease hippocampus. Front. Cell. Infect. Microbiol..

[71] Friedland R.P., McMillan J.D., Kurlawala Z. (2020). What are the molecular mechanisms by which functional bacterial amyloids influence amyloid beta deposition and neuroinflammation in neurodegenerative disorders?. Int. J. Mol. Sci..

[72] Jana M., Palencia C.A., Pahan K. (2008). Fibrillar amyloid-beta peptides activate microglia via TLR2: Implications for Alzheimer’s disease. J. Immunol..

[73] Jin J., Xu Z., Zhang L., Zhang C., Zhao X., Mao Y., Zhang H., Liang X., Wu J., Yang Y. (2023). Gut-derived β-amyloid: Likely a centerpiece of the gut-brain axis contributing to Alzheimer’s pathogenesis. Gut Microbes.

[74] Friedland R.P., Chapman M.R. (2017). The role of microbial amyloid in neurodegeneration. PLoS Pathog..

[75] Liu M., He C., Lv X., Zheng W., Yin J., Li Y., Ma J. (2026). Bile acid signaling mediates gut microbiota regulation of host lipid metabolism. Front. Microbiol..

[76] Sabahat S.E., Saqib M., Talib M., Shaikh T.G., Khan T., Kailash S.J. (2024). Bile acid modulation by gut microbiota: A bridge to understanding cognitive health. Ann. Med. Surg..

[77] Ehtezazi T., Rahman K., Davies R., Leach A.G. (2023). The pathological effects of circulating hydrophobic bile acids in Alzheimer’s disease. J. Alzheimer’s Dis. Rep..

[78] Tettevi E.J., Simpong D.L., Maina M., Adjei S., Asuming-Brempong E., Osei-Atweneboana M.Y., Ocloo A. (2025). Antibiotic-induced gut dysbiosis modulates Alzheimer’s disease-associated gene expression and protein aggregation in 3xTg-AD mice via the gut-brain axis. Brain Behav..

[79] Xie J., Van Hoecke L., Van Wonterghem E., Van Imschoot G., Andries V., Vereecke L., Vandenbroucke R.E. (2025). The gut-brain axis in Alzheimer’s disease is shaped by commensal gut microbiota-derived extracellular vesicles. Gut Microbes.

[80] Benameur T., Hasan A., Toufig H., Panaro M.A., Filannino F.M., Porro C. (2025). Microbiota-derived extracellular vesicles as potential mediators of gut-brain communication in traumatic brain injury: Mechanisms, biomarkers, and therapeutic implications. Biomolecules.

[81] Wasén C., Beauchamp L.C., Vincentini J., Li S., LeServe D.S., Gauthier C., Lopes J.R., Moreira T.G., Ekwudo M.N., Yin Z. (2024). Bacteroidota inhibit microglia clearance of amyloid-beta and promote plaque deposition in Alzheimer’s disease mouse models. Nat. Commun..

[82] Czaj P.V., Szewczyk-Golec K., Nuszkiewicz J., Woźniak A. (2026). Gut dysbiosis and microbiota-derived metabolites in neurodegenerative diseases: Molecular and biochemical mechanisms along the gut-brain axis. Molecules.

[83] Ren J., Wang Q., Hong H., Tang C. (2025). Fecal microbiota transplantation in Alzheimer’s disease: Mechanistic insights through the microbiota-gut-brain axis and therapeutic prospects. Microorganisms.

[84] Park S.-H., Lee J.-H., Kim J.-S., Kim T.J., Shin J., Im J.H., Cha B., Lee S., Kwon K.S., Shin Y.W. (2022). Fecal microbiota transplantation can improve cognition in patients with cognitive decline and Clostridioides difficile infection. Aging.

[85] Kim J.-S., Park H., Lee J.-H., Shin J., Cha B., Kwon K.S., Shin Y.W., Kim Y., Kim Y., Bae J.S. (2024). Effect of altered gene expression in lipid metabolism on cognitive improvement in patients with Alzheimer’s dementia following fecal microbiota transplantation: A preliminary study. Ther. Adv. Neurol. Disord..

[86] Nagpal R., Neth B.J., Wang S., Mishra S.P., Craft S., Yadav H. (2020). Gut mycobiome and its interaction with diet, gut bacteria and Alzheimer’s disease markers in subjects with mild cognitive impairment: A pilot study. eBioMedicine.

[87] Zhu Z., Ma X., Wu J., Xiao Z., Wu W., Ding S., Zheng L., Liang X., Luo J., Ding D. (2022). Altered gut microbiota and its clinical relevance in mild cognitive impairment and Alzheimer’s disease: Shanghai Aging Study and Shanghai Memory Study. Nutrients.

[88] Li J., Yuan Z., Li J., Liu Z., Wang Y., Cui M., Suo C., Jin L., Ding D., Chen X. (2025). Immune, blood-brain barrier, and metabolic biomarkers mediate gut-brain axis crosstalk in Alzheimer’s disease. Biomark. Res..

[89] Manfredi J.N., Gupta S.K., Vyavahare S., Deak F., Lu X., Buddha L., Wankhade U., Lohakare J., Isales C., Fulzele S. (2025). Gut microbiota dysbiosis in Alzheimer’s disease: Insights from human clinical studies and the mouse Alzheimer’s disease models. Physiol. Behav..

[90] de J.R. De-Paula V., Forlenza A.S., Forlenza O.V. (2018). Relevance of gut microbiota in cognition, behaviour and Alzheimer’s disease. Pharmacol. Res..

[91] Sochocka M., Donskow-Łysoniewska K., Diniz B.S., Kurpas D., Brzozowska E., Leszek J. (2019). The gut microbiome alterations and inflammation-driven pathogenesis of Alzheimer’s disease-a critical review. Mol. Neurobiol..

[92] Durairajan S.S.K., Singh A.K., Sulaiman S.M., Patnaik S., Krishnamoorthi S., Iyaswamy A., Vellingiri B., Yang C.-B., Williams L.L. (2025). Molecular links between inflammatory bowel disease and Alzheimer’s disease through immune signaling and inflammatory pathways. World J. Gastroenterol..

[93] Tönnies E., Trushina E. (2017). Oxidative stress, synaptic dysfunction, and Alzheimer’s disease. J. Alzheimer’s Dis..

[94] Guo T., Noble W., Hanger D.P. (2017). Roles of tau protein in health and disease. Acta Neuropathol..

[95] Area-Gomez E., Schon E.A. (2016). Mitochondria-associated ER membranes and Alzheimer disease. Curr. Opin. Genet. Dev..

[96] Yano J.M., Yu K., Donaldson G.P., Shastri G.G., Ann P., Ma L., Nagler C.R., Ismagilov R.F., Mazmanian S.K., Hsiao E.Y. (2015). Indigenous bacteria from the gut microbiota regulate host serotonin biosynthesis. Cell.

[97] Hou Y., Li J., Ying S. (2023). Tryptophan metabolism and gut microbiota: A novel regulatory axis integrating the microbiome, immunity, and cancer. Metabolites.

[98] Guillemin G.J., Brew B.J. (2002). Implications of the kynurenine pathway and quinolinic acid in Alzheimer’s disease. Redox Rep..

[99] Bravo J.A., Forsythe P., Chew M.V., Escaravage E., Savignac H.M., Dinan T.G., Bienenstock J., Cryan J.F. (2011). Ingestion of Lactobacillus strain regulates emotional behavior and central GABA receptor expression in a mouse via the vagus nerve. Proc. Natl. Acad. Sci. USA.

[100] Tang W., Meng Z., Li N., Liu Y., Li L., Chen D., Yang Y. (2021). Roles of gut microbiota in the regulation of hippocampal plasticity, inflammation, and hippocampus-dependent behaviors. Front. Cell Infect. Microbiol..

[101] Zhang Y., Zhang J., Wang Y., Yao J. (2023). Global trends and prospects about synaptic plasticity in Alzheimer’s disease: A bibliometric analysis. Front. Aging Neurosci..

[102] Ogbonnaya E.S., Clarke G., Shanahan F., Dinan T.G., Cryan J.F., O’Leary O.F. (2015). Adult hippocampal neurogenesis is regulated by the microbiome. Biol. Psychiatry.

[103] Bairamian D., Sha S., Rolhion N., Sokol H., Dorothée G., Lemere C.A., Krantic S. (2022). Microbiota in neuroinflammation and synaptic dysfunction: A focus on Alzheimer’s disease. Mol. Neurodegener..

[104] Mayer E.A., Nance K., Chen S. (2022). The gut-brain axis. Annu. Rev. Med..

[105] Parker A., Romano S., Ansorge R., Aboelnour A., Le Gall G., Savva G.M., Pontifex M.G., Telatin A., Baker D., Jones E. (2022). Fecal microbiota transfer between young and aged mice reverses hallmarks of the aging gut, eye, and brain. Microbiome.

[106] Gupta P.S., Padmakumar V.M., Usmani O.I. (2025). Cross-sectional analysis of gut microbiome diversity with progression of Alzheimer’s disease. Bioinformation.

[107] Tamtaji O.R., Heidari-Soureshjani R., Mirhosseini N., Kouchaki E., Bahmani F., Aghadavod E., Tajabadi-Ebrahimi M., Asemi Z. (2019). Probiotic and selenium co-supplementation, and the effects on clinical, metabolic and genetic status in Alzheimer’s disease: A randomized, double-blind, controlled trial. Clin. Nutr..

[108] Scarmeas N., Stern Y., Tang M.X., Mayeux R., Luchsinger J.A. (2006). Mediterranean diet and risk for Alzheimer’s disease. Ann. Neurol..

[109] Sofi F., Abbate R., Gensini G.F., Casini A. (2010). Accruing evidence on benefits of adherence to the Mediterranean diet on health: An updated systematic review and meta-analysis. Am. J. Clin. Nutr..

[110] Morris M.C., Tangney C.C., Wang Y., Sacks F.M., Bennett D.A., Aggarwal N.T. (2015). MIND diet associated with reduced incidence of Alzheimer’s disease. Alzheimer’s Dement..

[111] Solch R.J., Aigbogun J.O., Voyiadjis A.G., Talkington G.M., Darensbourg R.M., O’Connell S., Pickett K.M., Perez S.R., Maraganore D.M. (2022). Mediterranean diet adherence, gut microbiota, and Alzheimer’s or Parkinson’s disease risk: A systematic review. J. Neurol. Sci..

[112] Hoscheidt S., Sanderlin A.H., Baker L.D., Jung Y., Lockhart S., Kellar D., Whitlow C.T., Hanson A.J., Friedman S., Register T. (2022). Mediterranean and Western diet effects on Alzheimer’s disease biomarkers, cerebral perfusion, and cognition in mid-life: A randomized trial. Alzheimer’s Dement..

[113] Ghosh T.S., Rampelli S., Jeffery I.B., Santoro A., Neto M., Capri M., Giampieri E., Jennings A., Candela M., Turroni S. (2020). Mediterranean diet intervention alters the gut microbiome in older people reducing frailty and improving health status: The NU-AGE 1-year dietary intervention across five European countries. Gut.

[114] Gardener S.L., Rainey-Smith S.R., Barnes M.B., Sohrabi H.R., Weinborn M., Lim Y.Y., Harrington K., Taddei K., Gu Y., Rembach A. (2015). Dietary patterns and cognitive decline in an Australian study of ageing. Mol. Psychiatry.

[115] Singh N.A., Bhardwaj V., Ravi C., Ramesh N., Mandal A.K.A., Khan Z.A. (2018). EGCG Nanoparticles Attenuate Aluminum Chloride Induced Neurobehavioral Deficits, Beta Amyloid and Tau Pathology in a Rat Model of Alzheimer’s Disease. Front. Aging Neurosci..

[116] Gibson G.R., Hutkins R., Sanders M.E., Prescott S.L., Reimer R.A., Salminen S.J., Scott K., Stanton C., Swanson K.S., Cani P.D. (2017). Expert consensus document: The International Scientific Association for Probiotics and Prebiotics (ISAPP) consensus statement on the definition and scope of prebiotics. Nat. Rev. Gastroenterol. Hepatol..

[117] Dalile B., Van Oudenhove L., Vervliet B., Verbeke K. (2019). The role of short-chain fatty acids in microbiota-gut-brain communication. Nat. Rev. Gastroenterol. Hepatol..

[118] Hoffman J.D., Yanckello L.M., Chlipala G., Hammond T.C., McCulloch S.D., Parikh T., Sun S., Morganti J.M., Green S.J., Lin A.L. (2019). Dietary inulin alters the gut microbiome, enhances systemic metabolism and reduces neuroinflammation in an APOE4 mouse model. PLoS ONE.

[119] Henderson S.T., Vogel J.L., Barr L.J., Garvin F., Jones M.N., Costantini L.C. (2009). Study of the ketogenic agent AC-1202 in mild to moderate Alzheimer’s disease: A randomized, double-blind, placebo-controlled, multicenter trial. Nutr. Metab..

[120] Augustin K., Khabbush A., Williams S., Eaton S., Orford M., Cross J.H., Heales S.J.R., Walker M.C., Williams R.S.B. (2018). Mechanisms of action for the medium-chain triglyceride ketogenic diet in neurological and metabolic disorders. Lancet Neurol..

[121] Dupuis N., Curatolo N., Benoist J.F., Auvin S. (2015). Ketogenic diet exhibits anti-inflammatory properties. Epilepsia.

[122] Hill C., Guarner F., Reid G., Gibson G.R., Merenstein D.J., Pot B., Morelli L., Canani R.B., Flint H.J., Salminen S. (2014). Expert consensus document: The International Scientific Association for Probiotics and Prebiotics consensus statement on the scope and appropriate use of the term probiotic. Nat. Rev. Gastroenterol. Hepatol..

[123] Bonfili L., Cecarini V., Cuccioloni M., Angeletti M., Berardi S., Scarpona S., Rossi G., Eleuteri A.M. (2018). SLAB51 Probiotic Formulation Activates SIRT1 Pathway Promoting Antioxidant and Neuroprotective Effects in an AD Mouse Model. Mol. Neurobiol..

[124] Wang Q.-J., Shen Y.-E., Wang X., Fu S., Zhang X., Zhang Y.-N., Wang R.-T. (2020). Concomitant memantine and Lactobacillus plantarum treatment attenuates cognitive impairments in APP/PS1 mice. Aging.

[125] Plovier H., Everard A., Druart C., Depommier C., Van Hul M., Geurts L., Chilloux J., Ottman N., Duparc T., Lichtenstein L. (2017). A purified membrane protein from Akkermansia muciniphila or the pasteurized bacterium improves metabolism in obese and diabetic mice. Nat. Med..

[126] Li X., Lv C., Song J., Li J. (2021). Effect of Probiotic Supplementation on Cognitive Function and Metabolic Status in Mild Cognitive Impairment and Alzheimer’s Disease: A Meta-Analysis. Front. Nutr..

[127] Kim C.S., Cha J., Sim M., Jung S., Chun W.Y., Baik H.W., Shin D.M. (2021). Probiotic supplementation improves cognitive function and mood with changes in gut microbiota in community-dwelling older adults: A randomized, double-blind, placebo-controlled, multicenter trial. J. Gerontol. A Biol. Sci. Med. Sci..

[128] Lew L.-C., Hor Y.-Y., Yusoff N.A.A., Choi S.-B., Yusoff M.S., Roslan N.S., Ahmad A., Mohammad J.A., Abdullah M.F.I., Zakaria N. (2019). Probiotic Lactobacillus plantarum P8 alleviated stress and anxiety while enhancing memory and cognition in stressed adults: A randomised, double-blind, placebo-controlled study. Clin. Nutr..

[129] van Nood E., Vrieze A., Nieuwdorp M., Fuentes S., Zoetendal E.G., de Vos W.M., Visser C.E., Kuijper E.J., Bartelsman J.F., Tijssen J.G. (2013). Duodenal infusion of donor feces for recurrent Clostridium difficile. N. Engl. J. Med..

[130] Hazan S. (2020). Rapid improvement in Alzheimer’s disease symptoms following fecal microbiota transplantation: A case report. J. Int. Med. Res..

[131] ClinicalTrials.gov (2025). Clinical Safety and Efficacy of Fecal Microbiota Transplantation (FMT) in the Treatment of Alzheimer’s Disease (FMT-AD).

[132] ClinicalTrials.gov (2020). Oral Fecal Microbiota Transplant Feasibility Study in Alzheimer’s Disease; Identifier: NCT03998423; Alzheimer’s Disease and Faecal Microbiota Transplantation—A Pilot Study; Identifier: NCT07332260.

[133] Govindarajan N., Agis-Balboa R.C., Walter J., Sananbenesi F., Fischer A. (2011). Sodium butyrate improves memory function in an Alzheimer’s disease mouse model when administered at an advanced stage of disease progression. J. Alzheimer’s Dis..

[134] Schwarcz R., Bruno J.P., Muchowski P.J., Wu H.Q. (2012). Kynurenines in the mammalian brain: When physiology meets pathology. Nat. Rev. Neurosci..

[135] Maddison D.C., Giorgini F. (2015). The kynurenine pathway and neurodegenerative disease. Semin. Cell Dev. Biol..

[136] Kruger K., van der Veen H., Beigy M., O’Donovan S.D., van Riel N., Remie L.B., Aarts E., Steegenga W., Smeets P.A.M., van Trijp M.P.H. (2026). Gut-brain health effects of PREbiotics in older adults with suspected COgnitive DEcline: Design of the PRECODE randomised placebo-controlled trial. Front. Nutr..

[137] Cummings J.L., Atri A., Feldman H.H., Hansson O., Sano M., Knop F.K., Johannsen P., León T., Scheltens P. (2025). Evoke and evoke+: Design of two large-scale, double-blind, placebo-controlled, phase 3 studies evaluating efficacy, safety, and tolerability of semaglutide in early-stage symptomatic Alzheimer’s disease. Alzheimer’s Res. Ther..

[138] Cummings J.L., Atri A., Sano M., Zetterberg H., Scheltens P., Knop F.K., Johannsen P., Wichmann C.A., Abschneider R.M., Leon T. (2026). Efficacy and safety of oral semaglutide 14 mg (flexible dose) in early-stage symptomatic Alzheimer’s disease (evoke and evoke+): Two phase 3, randomised, placebo-controlled trials. Lancet.

[139] Johnson A.J., Vangay P., Al-Ghalith G.A., Hillmann B.M., Ward T.L., Shields-Cutler R.R., Kim A.D., Shmagel A.K., Syed A.N., Walter J. (2019). Daily sampling reveals personalized diet-microbiome associations in humans. Cell Host Microbe.

[140] Kraimi N., Ross T., Pujo J., De Palma G. (2024). The gut microbiome in disorders of gut-brain interaction. Gut Microbes.

[141] Donaldson A.I.C., Fyfe C.L., Martin J.C., Smith E.E., Horgan G.W., Myint P.K., Johnstone A.M., Scott K.P. (2025). Aging gut-brain interactions: Pro-inflammatory gut bacteria are elevated in fecal samples from individuals living with Alzheimer’s dementia. Geriatrics.

[142] Thevaranjan N., Puchta A., Schulz C., Naidoo A., Szamosi J., Verschoor C.P., Loukov D., Schenck L.P., Jury J., Foley K.P. (2017). Age-associated microbial dysbiosis promotes intestinal permeability, systemic inflammation, and macrophage dysfunction. Cell Host Microbe.

[143] Tran T.T.T., Corsini S., Kellingray L., Hegarty C., Le Gall G., Narbad A., Müller M., Tejera N., O’Toole P.W., Minihane A.-M. (2019). APOE genotype influences the gut microbiome structure and function in humans and mice: Relevance for Alzheimer’s disease pathophysiology. FASEB J..

[144] Dahl W.J., Rivero Mendoza D., Lambert J.M. (2020). Diet, nutrients and the microbiome. Prog. Mol. Biol. Transl. Sci..

[145] Mandal R., Paul T., Debnath R. (2026). Understanding the gut-brain axis with methodological challenges and transitional barriers in neurophysiology. Int. J. Nutr. Pharmacol. Neurol. Dis..

[146] Ou Y., Belzer C., Smidt H., de Weerth C. (2023). Methodological recommendations for human microbiota-gut-brain axis research. Microbiome Res. Rep..

[147] Quigley E.M. (2017). Gut microbiome as a clinical tool in gastrointestinal disease management: Are we there yet?. Nat. Rev. Gastroenterol. Hepatol..

[148] McDonald D., Hyde E., Debelius J.W., Morton J.T., Gonzalez A., Ackermann G., Aksenov A.A., Behsaz B., Brennan C., Chen Y. (2018). American gut: An open platform for citizen science microbiome research. mSystems.

[149] Lloyd-Price J., Arze C., Ananthakrishnan A.N., Schirmer M., Avila-Pacheco J., Poon T.W., Andrews E., Ajami N.J., Bonham K.S., Brislawn C.J. (2019). Multi-omics of the gut microbial ecosystem in inflammatory bowel diseases. Nature.

[150] Hooks K.B., Konsman J.P., O’Malley M.A. (2019). Microbiota-gut-brain research: A critical analysis. Behav. Brain Sci..

[151] Nguyen T.L.A., Vieira-Silva S., Liston A., Raes J. (2015). How informative is the mouse for human gut microbiota research?. Dis. Models Mech..

[152] Drummond E., Wisniewski T. (2017). Alzheimer’s disease: Experimental models and reality. Acta Neuropathol..

[153] Kashyap P.C., Chia N., Nelson H., Segal E., Elinav E. (2017). Microbiome at the frontier of personalized medicine. Mayo Clin. Proc..

[154] Zeevi D., Korem T., Zmora N., Israeli D., Rothschild D., Weinberger A., Ben-Yacov O., Lador D., Avnit-Sagi T., Lotan-Pompan M. (2015). Personalized nutrition by prediction of glycemic responses. Cell.

[155] Sonnenburg J.L., Bäckhed F. (2016). Diet-microbiota interactions as moderators of human metabolism. Nature.

[156] Jiménez-Pareyón G.R., Cristóbal-Luna J.M., García-Martínez Y., Garfias-Noguez C., Ramírez-Damián M., Torres-Maravilla E., Sánchez-Pardo M.E. (2025). Psychobiotics at the frontiers of neurodegenerative and neuropsychiatric research. Microorganisms.

[157] Wörheide M.A., Krumsiek J., Kastenmüller G., Arnold M. (2021). Multi-omics integration in biomedical research-a metabolomics-centric review. Anal. Chim. Acta.

[158] Zhao Q., Gou C., Luo G., Dai S., Wang D., Zhang S., Wang F., Xu H., Han Y., Wang S. (2025). Combined multi-omics and brain pathology reveal novel biomarkers for Alzheimer’s disease. Sci. Rep..

[159] Trushina E., Mielke M.M. (2014). Recent advances in the application of metabolomics to Alzheimer’s disease. Biochim. Biophys. Acta.

[160] Oso T.A., Ahmed M.M., Okesanya O.J., Adebayo U.O., Obadeyi K.B., Othman Z.K., Lucero-Prisno D.E. (2026). Exploring the gut-brain-microbiome axis in Alzheimer’s disease: Integrating metagenomics, metabolomics, and artificial intelligence for next-generation biomarker discovery. J. Alzheimer’s Dis..

[161] Sperling R.A., Donohue M.C., Raman R., Sun C.-K., Yaari R., Holdridge K., Siemers E., Johnson K.A., Aisen P.S., for the A4 Study Team (2020). Association of factors with elevated amyloid burden in clinically normal older individuals. JAMA Neurol..

[162] Oosthoek M., Vermunt L., de Wilde A., Bongers B., Antwi-Berko D., Scheltens P., van Bokhoven P., Vijverberg E.G.B., Teunissen C.E. (2024). Utilization of fluid-based biomarkers as endpoints in disease-modifying clinical trials for Alzheimer’s disease: A systematic review. Alzheimer’s Res. Ther..

